# SIMBA: Online Simulation for Teaching Medical Cases to Preclinical Students—A Pilot Study

**DOI:** 10.1111/tct.70070

**Published:** 2025-03-17

**Authors:** Aditya Swaminathan, Dengyi Zhou, Isabel Allison, Tamzin Ogiliev, Fatema Rezai, Georgia Morgan, Haaziq Sheikh, Farah Abdelhameed, Harjeet Kaur, Alice Yip, Catherine Cooper, Meri Davitadze, Eka Melson, Paul A. Foster, Vivek Dhir, SIMBA and CoMICs team, Punith Kempegowda

**Affiliations:** ^1^ College of Medical and Dental Sciences University of Birmingham Birmingham UK; ^2^ London North West University Healthcare NHS Trust London UK; ^3^ Faculty of Health University of Plymouth Plymouth UK; ^4^ Princess of Wales Hospital, Cwm Taf Morgannwg University Health Board Bridgend UK; ^5^ University of Warwick Coventry UK; ^6^ Walsall Manor Hospital, Walsall Healthcare NHS Trust Walsall UK; ^7^ Clinic NeoLab Tbilisi Georgia; ^8^ University of Leicester Leicester UK; ^9^ Department of Metabolism and Systems Sciences, College of Medical and Dental Sciences University of Birmingham Birmingham UK; ^10^ Institute of Clinical Sciences, College of Medical and Dental Sciences University of Birmingham Birmingham UK; ^11^ Department of Applied Health Sciences College of Medical and Dental Sciences Birmingham UK; ^12^ University Hospitals Birmingham NHS Foundation Trust Birmingham UK

**Keywords:** E‐learning, medical and pharmacy students, scenario‐based learning, simulation learning, small‐group teaching

## Abstract

**Background:**

Medical education employs diverse teaching strategies, including blending lecture‐based learning, small‐group teaching (SGT) and, increasingly, simulation‐based learning. Nonetheless, limitations in clinical application and participation persist. Simulation via Instant Messaging for Bedside Application (SIMBA) complements these methods by simulating real‐world clinical scenarios. This pilot study compares SIMBA's effectiveness with SGT in endocrine topics for medical and pharmacy students.

**Methods:**

The SIMBA for students model was developed using Kern's six‐step framework. SIMBA sessions, facilitated by trained moderators and senior experts, simulated outpatient consultations via WhatsApp. The study included SIMBA and SGT sessions from October 2020 to March 2022. Teaching effectiveness was assessed through postsession surveys and multiple‐choice questions (MCQs). The study compared the MCQ scores and student satisfaction of SIMBA, SGT and combined SIMBA + SGT cohorts.

**Results:**

One hundred thirty (103 medical and 27 pharmacy) students participated in 14 SIMBA sessions, and 150 students responded to the post‐SGT survey, with 38 attending both. Median MCQ scores were higher post‐SIMBA (75.0%) compared with post‐SGT (60.0%) (*p* < 0.0001). No significant difference was observed between SIMBA and SIMBA + SGT scores or SGT and SIMBA + SGT scores. SIMBA sessions were perceived as enjoyable (89.2%), intelligible (90.8%), engaging (81.5%), promoted new knowledge (90.0%) and enhanced comprehension (93.9%). 83.1% of students desired SIMBA to complement SGT.

**Conclusions:**

SIMBA demonstrated superior knowledge gain and student satisfaction compared to SGT. Its familiar technology and interactive format suit modern learning, offering a standardised and equitable experience. Integrating SIMBA into the curriculum could help overcome teaching limitations and better prepare students for clinical practice.

**Abbreviations**
LBL: lecture‐based learning
MCQs: multiple‐choice questions
SBL: simulation‐based learning
SGT: small‐group teaching
SIMBA: Simulation via Instant Messaging for Bedside Application

## 1 Background

Although lecture‐based learning (LBL) remains a fundamental component of medical education, modern curricula increasingly seek to integrate clinical exposure during the preclinical years using various methods [1, 2, 3]. Among these methods, scenario‐based learning has been recognised for bridging basic and clinical sciences in the preclinical medical curriculum [4, 5]. To facilitate this, many medical schools introduced small‐group teaching (SGT) [6, 7]. SGT, typically provided after lectures, involves students working in small groups of 5–10 to enhance interest, knowledge retention and communication skills [6, 7]. However, depending on the facilitators' experience, expertise, enthusiasm and students' engagement, this can result in differential knowledge gain [8]. Additionally, the scalability of SGT is often limited by time and resource constraints. Active participation can also have adverse effects, such as pressure on students to participate in group activities [7, 9].

Simulation‐based learning (SBL) has gained prominence as an educational approach aiming to provide immersive practical experiences for learners to develop skills, solve problems or understand complex concepts in a safe and realistic setting [3, 10]. SBL augments clinical performance, attitudes, and teamwork, ultimately helping improve patient outcomes [11]. Several studies have highlighted the superiority of SBL to LBL in training adaptability, situation awareness, critical assessment and management [12, 13, 14]. SBL enhances interpretation and knowledge retention compared with watching recorded content [15]. Concurrently, technological advancements have seen SBL increasingly incorporated into virtual platforms as an educational strategy to bridge gaps in accessibility, cost and effectiveness [16].

SBL augments clinical performance, attitudes and teamwork, ultimately helping improve patient outcomes [11]

Simulation via Instant Messaging for Bedside Application (SIMBA) is a novel scenario‐based e‐simulation approach using WhatsApp. Using SBL techniques, the approach aims to increase participants' confidence in clinical scenarios [17]. SIMBA for students simulates a virtual environment where preclinical medical and pharmacy students sit in with a consultant at an outpatient consultation, akin to a clinical observership they undergo as part of their medical school, building their confidence and priming them towards clinical placement. LBL and case‐based discussion (CBD) are well‐established teaching methods that provide efficient knowledge delivery and introduce complex concepts. As such, they form an integral part of the broader SIMBA model (Figure 3). In SIMBA for students, the simulation‐based component serves as an adjunct to these traditional methods, offering a more immersive and experiential learning approach. Unlike LBL and CBD, where knowledge is introduced before application, SIMBA reverses this sequence—participants engage in a live, interactive scenario before receiving instantaneous feedback and reflection. This method enhances integrative learning, improves adaptability, and strengthens decision‐making in a safe, controlled environment [17]. SIMBA helps bridge the gap between theory and practice by building confidence through hands‐on experience. However, CBD remains valuable for fostering critical thinking through structured analysis and discussion. To reinforce this, postsimulation debriefing with a senior expert follows a CBD‐style approach, enabling participants to reflect on their clinical reasoning, decision‐making and key learning points from the scenario. The SIMBA model is grounded in research, utilising gaming simulation design [18] and Kolb's experiential learning theory [19]. The educational strategy incorporates elements of interprofessional education, uses minimal resources and sustained medical education even during the pandemic [20, 21]. Previous sessions have focused on postgraduate healthcare professionals demonstrating that SIMBA is an effective teaching model that leads to sustained improvements in clinical knowledge [17, 22, 23].

Simulation via Instant Messaging for Bedside Application (SIMBA) is a novel scenario‐based e‐simulation approach using WhatsApp

This pilot study aims to report on the feasibility of adopting the SIMBA model to deliver applied learning to undergraduate medical and pharmacy students using the six‐step conceptual framework described by Kern et al. [24]. The acceptance and objective performance of preclinical students post‐SIMBA in comparison with SGT is reported.

## 2 Materials and Methods

**FIGURE 1:**
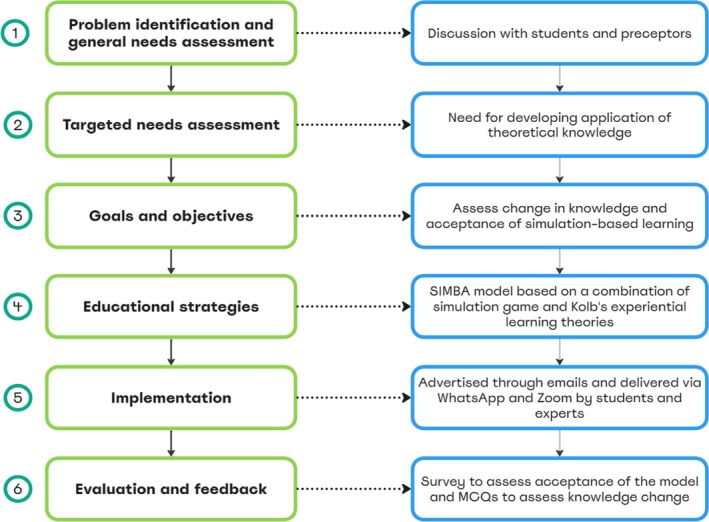
The six‐step conceptual framework adopted to deliver SIMBA for students based on Kern et al.

The SIMBA for students model was developed from October 2020 to March 2022, employing Kern's six‐step conceptual framework [24, 25] (Figure 1). This process is outlined below.

### 2.1 Problem Identification and General Needs Assessment

The Institute of Metabolism and Systems Research, University of Birmingham, delivers endocrine‐related SGT to Year 1 and 2 medical and pharmacy students. At the University of Birmingham, group sizes are usually 16. SGT sessions typically involve a discussion of 5–7‐min of four to five cases, each with two to three questions. Meetings with the institute's medical education director and his team were organised to evaluate the strengths and limitations of the current SGT model. Discussions revealed that SGT often involved differential active participation due to inconsistent group dynamics and varied student preparedness. This may exacerbate the differential gain from SGT.

### 2.2 Targeted Needs Assessment

**FIGURE 2:**
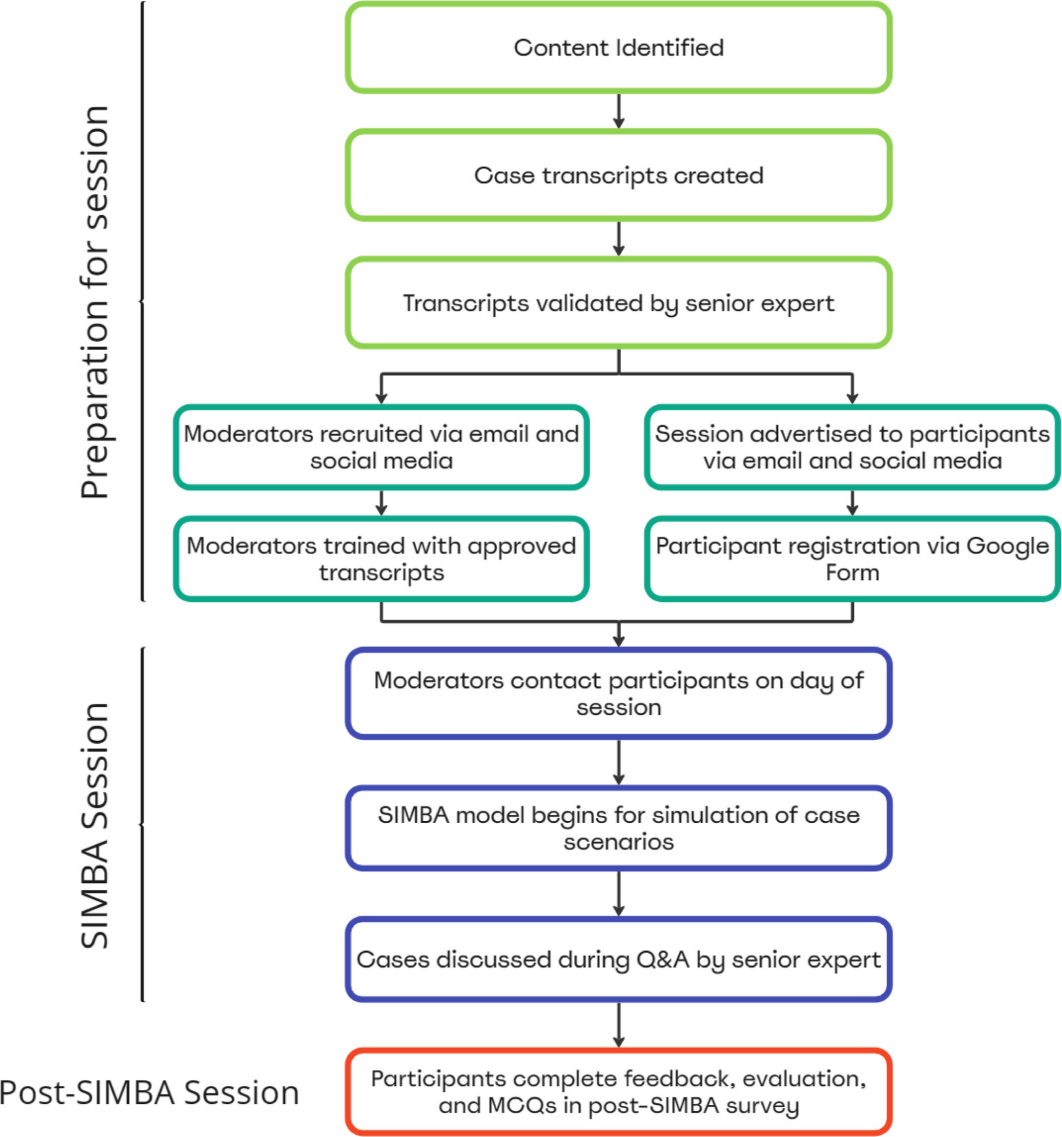
The implementation phase of the six‐step conceptual framework based on Kern et al. describing the working model of SIMBA for students.

Following discussions with medical students (who form the end user cohort), we identified a need for a model that provides more uniform active participation from students while ensuring standardised applied learning was identified. SIMBA's success in postgraduate learning [17] provided a platform to deliver a similar model for undergraduate students as an alternative to SGT. A working model was finalised following several discussions with the teams responsible for delivering endocrine‐related lectures and SGTs for undergraduate medical and pharmacy students (Figure 2).

### 2.3 Goals and Objectives

We aimed to develop a model based on SIMBA that would improve engagement, stimulate interest, use modest resources, and result in a minimum additional workload for students and teachers. We assessed students' change in knowledge with SIMBA compared with SGT and acceptance of SIMBA for students as an educational model.

We aimed to develop a model based on SIMBA that would improve engagement, stimulate interest, use modest resources, and result in a minimum additional workload for students and teachers.

### 2.4 Education Strategies

**FIGURE 3:**
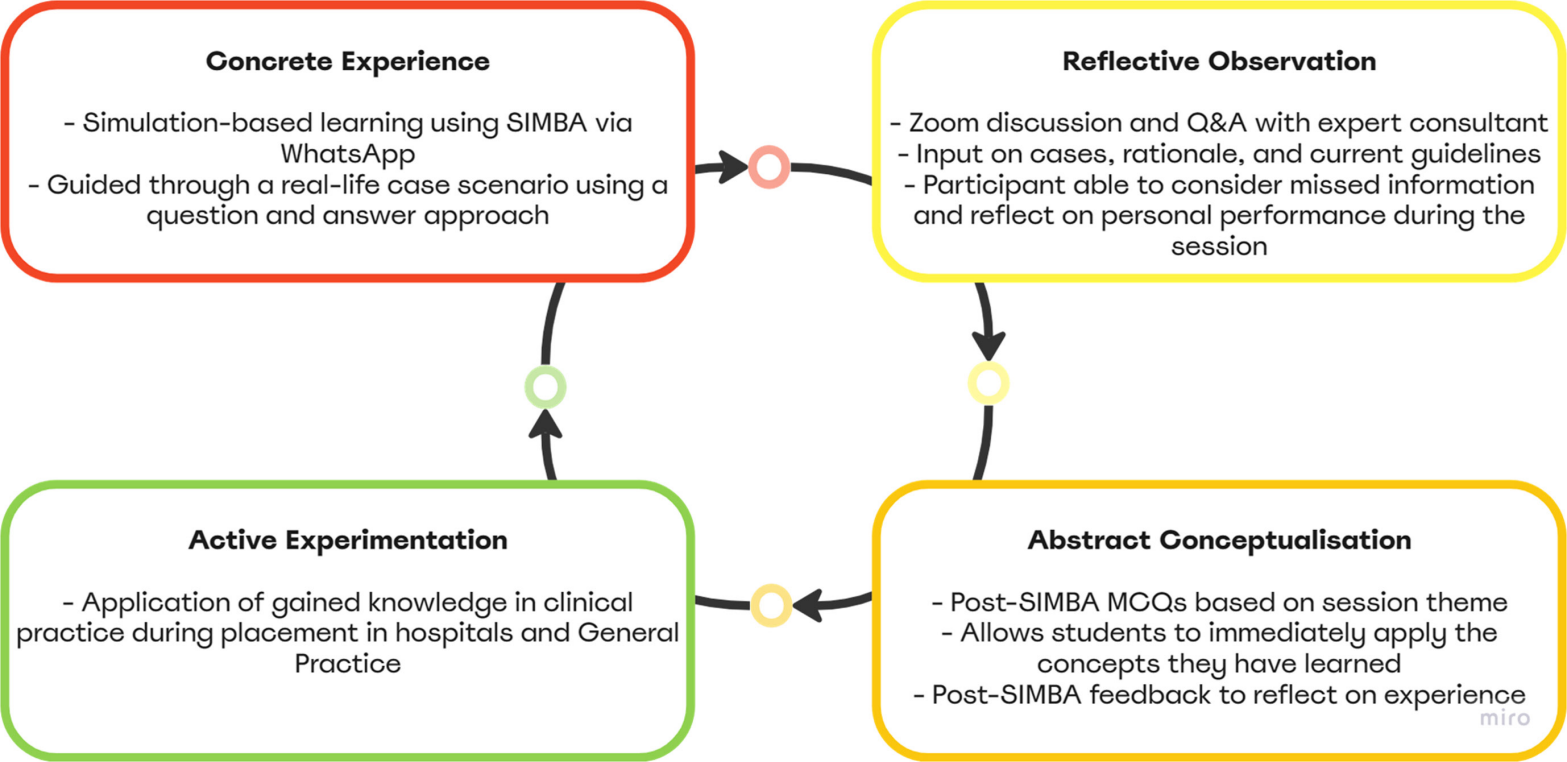
Visualisation of SIMBA for students' educational strategy based on Kolb's experiential learning theory.

The SIMBA for students model combines the simulation game and Kolb's experiential learning theories [18, 19]. A simulation game is defined as the effects of decisions made by participants interrelated with a system of rules and references to resources that realistically symbolise reality [18]. Figure 3 demonstrates the educational strategy of SIMBA for students using Kolb's four‐stage experiential learning cycle [19].

### 2.5 Implementation of the Model

The SIMBA for students team was established following the model of the SIMBA steering committee. The process began with advertising the available roles (case design lead, moderator recruitment lead, advertising lead, and forms and surveys lead), followed by conducting a baseline survey of interested candidates and subsequent interviews. The selection panel included a consultant supervisor and several senior medical students. Once selected, the team members shadowed the SIMBA steering committee to comprehensively understand their roles and responsibilities before commencing independent work in SIMBA for students. After the team was formed, a lead for each session was designated. The session lead was responsible for coordinating with various stakeholders, including the year's teaching program director and the clinical expert (consultant clinician) delivering the lecture, to identify and schedule a suitable date for the session.

The SIMBA for students team prepared standardised transcripts for adrenal, diabetes, metabolic bone, thyroid, and reproductive endocrinology sessions based on content in lectures delivered to students. Each transcript included three case scenarios, each with 10 questions exploring a topic relating to the overarching theme of the session. The SIMBA model simulates students placed in an outpatient setting in general practice or hospital placement alongside a clinician. Participants undergo a series of conversations to explore the presentation, pathophysiology, and approach to the clinical scenario in greater depth. Experts in the relevant field, typically consultants, validated all transcripts to ensure accuracy. The transcript would be a template for student moderators to deliver the case scenarios to the participants during the session.

A student session lead, a senior expert, and student moderators facilitated each SIMBA session. All moderators were trained in advance using the finalised transcripts to ensure efficiency and accuracy during the session. Standardised transcripts and training processes ensured that equal experiences were delivered to all participants.

Sessions were voluntary and advertised to students via cohort‐wide university emails, and information was further cascaded to relevant groups by fellow students through social media, including WhatsApp and Instagram. Students interested in participating completed informed consent and registered via a Google Form to participate in the session. Forms were closed 24 h before the session, and all participants were allocated a moderator. Participant numbers ranged from 10 to 80 per session. Each moderator was responsible for up to five participants, depending on their experience in moderating.

Although SIMBA sessions required more moderators than SGT—three to four for a group of 16 compared with just one tutor—these moderators were senior students (e.g., Year 2 students moderated sessions for Year 1). This allowed the senior students to revise early‐year content and ensured uniform learning for all participants, as they followed a standard script. Training for moderators involves a one‐time 30‐min session with a SIMBA for students' team members. The training included familiarising with transcripts and session structure and communicating appropriately with participants by practising a ‘mock’ session.

Moderators were provided with the assigned participants' anonymised SIMBA IDs and WhatsApp numbers. On the day of the session, moderators introduced themselves to participants via standardised text 1 h before the session. Following this, moderators shared the agenda for the session via WhatsApp. Five minutes before the session, a Zoom link for the ‘Opening Welcome’ was sent to all participants. In the ‘Opening Welcome’ Zoom call, students were introduced to SIMBA and what the session involved. Following this, moderators sent an image as a signal to start the simulation (Data S1). Simulations were initiated once the participants confirmed they were ready.

There were three cases per session, with a 2‐min break between each case. The moderators interacted with participating students and provided them with appropriate scenarios followed by questions. Once the students answered the questions, the moderators provided them with model answers. Each case simulation lasted 15 min and was stopped after this time, regardless of whether the participant completed all questions. If the participant did not complete the simulation in the stipulated time, the remaining content was provided to the student at the end of the session for personal review. At the end of each case, students were asked if they had any queries. All comments were compiled and shared with the expert leading the discussion.

After the simulation, participants were invited to Zoom for discussion. The senior expert summarised salient learning points for each case, followed by an opportunity for participants to ask questions about the case or general questions related to the topic. This lasted 10 min for each case.

### 2.6 Evaluation, Feedback, Data Collection and Handling

We adopted a mixed‐methods approach with a concurrent embedded design for data collection using the post‐SIMBA survey to ensure comprehensive and meaningful responses. Participants were invited to comment on various aspects of the simulation session using a 5‐point Likert scale and open‐ended free‐text questions. Ten MCQs, tailored to relevant learning objectives, were included to assess their knowledge after the SIMBA session. Seventy‐five percent of MCQs were designed to assess the basic recall of information, whereas the remaining 25% incorporated a two‐step single‐best‐answer approach to test higher‐order thinking skills (Data S2). To ensure the reliability and validity of MCQs, they are created by healthcare professionals and senior students familiar with the University's undergraduate curriculum. Following this, MCQs are validated by senior experts in the field and/or module leads who would otherwise typically be involved in producing comparable questions for summative university assessments. This ensures the questions are of a standardised level and similar to the quality of questions that a student may receive in an exam. All students in the year group were invited to complete a similar survey, including the same MCQs, following the related SGT organised and delivered by the University. The development of the post‐SIMBA survey follows the efforts of Melson et al., who initially piloted this survey in previous SIMBA sessions [17]. Following this, the survey has undergone several iterations to address feedback and adapt for delivery to an undergraduate student population. As described by Kelley et al., the design of a survey, with regard to its wording, form, content and more, can result in unintended bias within survey results [26]. Following this guidance, the post‐SIMBA survey was designed and iterated with several key features in mind, such as a clearly stated purpose, relevant and balanced question types, appropriate length, and adherence to data protection requirements.

Several measures were taken to minimise bias in data collection and handling. All surveys were anonymised during analysis, preventing any association between the participants' identities and responses. The analyst was blinded to the hypothesis to reduce confirmation bias. Strict protocols were followed for data cleaning and transformation, and all work was cross‐validated and peer‐reviewed. However, there may have been non‐response bias, as not everyone who attended the SGT sessions responded to the postsession survey.

## 3 Statistical Analysis

Survey responses were grouped among those students who attended SIMBA only, SGT only and both SIMBA and SGT (SIMBA + SGT). Descriptive statistics were expressed as a percentage, and comparative box and whisker diagrams were created using Stata version 17.0, which compared SIMBA only, SGT only and SIMBA + SGT MCQ scores. The difference in MCQ scores between groups was analysed using the Wilcoxon sign‐rank test. *p*‐values of < 0.05 were considered statistically significant.

Responses to open‐ended questions were analysed using single‐coder inductive thematic analysis. Material collected from both surveys was read and familiarised individually. Similar responses were grouped and systematically assigned labels based on the main points, and recurrent labels were collated into themes. The themes identified are presented in the analysis, including examples from post‐SIMBA and post‐SGT surveys, encompassing the variety of opinions expressed. Given the study's exploratory nature, the decision to rely on a single source of qualitative data ensured consistency and depth in thematic interpretation.

Data triangulation was not employed in this study because of the focus on capturing in‐depth insights specific to the postsession surveys collected as part of a concurrent embedded design. In this design, the qualitative data served a complementary role, enriching the primary focus of the study without requiring integration from additional data sources. Although the absence of triangulation may be considered a limitation, it was mitigated by the systematic approach taken during the analysis and the explicit acknowledgement of this methodological choice. The findings are presented transparently, with illustrative examples supporting the identified themes.

## 4 Results

Between October 2020 and March 2022, 130 students attended 14 SIMBA sessions. Twenty‐seven were Pharmacy students, with the remaining consisting of Year 1 and 2 MBChB students. After attending the corresponding SGT sessions, 150 students responded to the post‐SGT survey. Of these, 38 also attended the related SIMBA session.

**FIGURE 4:**
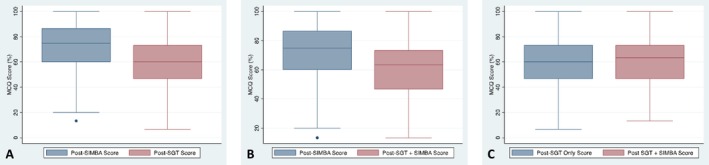
(a) Box and whisker diagram showing a comparison of MCQ scores between post‐SIMBA and post‐SGT only. (b) Box and whisker diagram showing a comparison of MCQ scores between students attending only SIMBA and those who attended both SIMBA and SGT. (c) Box and whisker diagram showing a comparison of MCQ scores between students who attended SGT only and those who attended both SGT and SIMBA.

Median (IQR) MCQ scores were 75.0% (60.0%–86.7%) post‐SIMBA, 60.0% (46.7%–73.3%) post‐SGT and 63.3% (46.7%–73.3%) for post‐SIMBA and SGT combined. Post‐SIMBA scores were significantly higher than post‐SGT only (*p* < 0.0001) (Figure 4a). There was no significant difference in MCQ scores between those who attended SIMBA only and those who attended both SIMBA and SGT (*p* = 0.0731) (Figure 4b). Similarly, there was no significant difference between students who attended SGT only and those who attended SGT and SIMBA (*p* = 0.0542) (Figure 4c).

Students enjoyed both SIMBA (89.2%) and SGT sessions (87.9%). Students who attended SIMBA thought that the session was easy to follow (90.8%), engaging and interactive (81.5%), promoted new knowledge (90.0%) and created a friendly environment to ask questions (84.6%). SIMBA stimulated more interest in endocrinology than SGT sessions (81.5% vs. 73.2%) and provided a more in‐depth understanding of topics covered than SGT sessions (93.9% vs. 83.9%). 63.1% of students thought the time allocated for each SIMBA case was sufficient, compared with 81.2% for each SGT case. 83.1% of students would like to have SIMBA as an adjunct to their usual SGT sessions, and overall, more students felt better prepared for the endocrine topic covered post‐SIMBA (86.9%) compared with post‐SGT (49.7%).

Students who attended SIMBA thought that the session was easy to follow (90.8%), engaging and interactive (81.5%), promoted new knowledge (90.0%) and created a friendly environment to ask questions (84.6%).

Thematic analysis of open‐ended questions revealed that SIMBA facilitated interactive learning, application of knowledge applicable to clinical scenarios, instant feedback and enjoyment in case‐based learning. SGT benefitted from working in small groups and the content acting as a peer discussion prompt (Data S3). Suggestions to improve SGT focused on the need for instantaneous feedback, increased tutor support and presence, as this was variable among groups, a clear structure and timing for sessions and clear guidance on prior preparation (Data S4). Suggestions for improvement of SIMBA focused on personalised moderator interaction, case content and session timing, suggesting both improvements to timing during the session and about the curriculum (Data S5).

## 5 Discussion

SIMBA demonstrated superior student satisfaction and improved MCQ performance compared with SGT. Attending SIMBA and SGT did not lead to more significant performance than SIMBA‐only, suggesting that SIMBA may be sufficient as a stand‐alone teaching modality.

Effective teaching models and environments must cater to the target audience's preferences. Younger generations favour technology‐assisted learning [27, 28]. SIMBA utilises technology familiar to this generation and provides teaching in an environment more conducive to learning by efficiently combining e‐learning and SBL.

Educational designers face challenges in selecting instructional design features, including determining the appropriate level of fidelity and type of structure [16]. The six‐step conceptual framework helped to define and address the needs, goals and objectives to deliver end‐user feedback‐driven SBL [24]. Although combining the simulation game and Kolb's experiential learning theories helped deliver an engaging session [18, 19], open‐ended feedback highlighted the need to ensure students attended the lectures before participation to maximise learning. Thus, the model has been revised to include lectures as part of the concrete experience. The active experimentation stage of our learning model could not be measured. A follow‐up interview of participants to explore how they use the knowledge gained from SIMBA for students during their interactions with patients at clinical placements can address this. Stimulating interest in participating was difficult as students had pre‐existing academic commitments. The effect of burnout on student performance is well‐documented [29]. This was considered during the design process. Scenario‐based learning uses briefer scenarios, unlike CBDs [30]. Thus, it aimed to combine scenario‐based learning with simulation, which has been stated to serve as an effective measure to fill educational gaps [16]. However, controlling and selecting appropriate levels of fidelity proved difficult. The level of fidelity makes minimal difference in learning transfer, but importantly, it can impact engagement and cost‐effectiveness (regarding both time and money) [16]. Olaussen et al. had their students actively perform a hands‐on simulation [31]. This high level of fidelity is perhaps better suited to the more advanced learner than those involved in this study [16]. Regardless, in this study, it can be argued that the balance of fidelity was reversed in the wrong direction, resulting in cases where the students adopted a level of detail that proved to be either a distraction for the student or too overwhelming. As discussed by Bahattab et al., developing an SBES is a challenging task requiring collaborative efforts [16]. To address this and to stimulate greater interest in future sessions, the structure of case scenarios needs to be revised to involve a more robust method of design that provides an increased focus on the scenario and its structure rather than simply aiming to address the learning objectives. However, creating such case studies can be a drawback for scenario‐based learning methods as it is time‐consuming [32]. However, this can be addressed with artificial intelligence and large language models.

The six‐step conceptual framework helped to define and address the needs, goals and objectives to deliver end‐user feedback‐driven SBL [24].

Interest is crucial in motivating learning, and the ability to stimulate interest in students is a powerful tool essential to academic success [33]. SIMBA stimulated greater interest than SGT, which may have contributed to the significant increase in MCQ scores after the SIMBA sessions.

Information becomes more valuable to students when contextualised and applicable to their goals [34]. Similar work elsewhere showed that virtual simulation tends to be well‐received, with several studies indicating student satisfaction and improved clinical confidence [31]. Nursing students participating in a virtual simulation teaching activity felt less pressured and less inhibited by faculty and peers during the session [35]. Unlike a real scenario, SBL provides a safe space to learn from mistakes, a critical aspect of the learning process. This sentiment was echoed by a study involving medical radiation sciences students, highlighting the importance of ‘safe practice in a low‐pressure environment’ [36]. Preliminary results from the obstetric and neonatal simulation workshop suggest it may be valuable to integrate interprofessional education into teaching curriculums post‐pandemic [37]. The involvement of pharmacy students as moderators in sessions aimed at medical students, and vice‐versa, incorporates an opportunity to reflect upon and understand the roles of other healthcare professionals.

Technology‐based learning tools can improve flexibility, adaptability, interactivity and cost efficiency [38]. Several studies highlight the benefits of SBL tools such as high‐fidelity mannequins, virtual reality, or augmented reality [39]. However, these technologies are often expensive or require costly subscriptions. SIMBA leverages accessible platforms like WhatsApp, reducing technical barriers and making SBL more cost‐effective and accessible. Requiring a smartphone and internet access, the model enables student participation across diverse geographical locations. Previous sessions have demonstrated its effectiveness in educating healthcare professionals worldwide [21, 22]. SIMBA is grounded in robust educational research, integrating Kolb's experiential learning theory and Kern's six‐step framework. This ensures a structured, evidence‐based approach. Unlike many e‐learning platforms that rely on pre‐recorded content or asynchronous discussions, SIMBA offers live, interactive sessions with trained moderators and expert‐led Q&A discussions. This real‐time engagement provides immediate, personalised feedback, enhancing the learning experience. Furthermore, each session is designed to align with the curriculum, ensuring direct relevance to students' learning needs and clinical practice. By combining accessibility, scalability and an interactive, research‐backed approach, SIMBA sets itself apart as an innovative and effective SBL model.

SIMBA leverages accessible platforms like WhatsApp, reducing technical barriers and making SBL more cost‐effective and accessible.

This pilot study focused on endocrinology, but the model can easily be expanded to any subject in the curriculum [40]. SIMBA has other advantages compared with SGT, including avoiding individual students dominating the discussion, shy or disinterested students failing to contribute and attention being directed towards the facilitator, who is expected to provide answers [7]. SIMBA is standardised and delivers equal experiences to all students taking part, thus avoiding the issue of differential knowledge gain depending on which facilitator is allocated when multiple parallel sessions are run. SIMBA also helps to develop peer‐to‐peer learning where older year students can revise concepts while moderating for younger years.

## 6 Limitations

Since participation in SIMBA is voluntary and not part of the formal curriculum, self‐selection bias may have drawn highly motivated students, potentially contributing to higher MCQ scores in SIMBA compared with SGT. Some students may have joined the session due to an interest in endocrinology or the unique style of e‐learning, thus leading to bias regarding whether the session stimulated interest in endocrinology or whether the session was engaging and interactive. Nonetheless, e‐learning may not be suitable for all learners. ‘Learner isolation’ can limit the overall effectiveness of a teaching programme [41, 42]. Technological issues, although typically resolvable, can also undermine the quality of an educational experience. Additionally, the limited number of students who participated in both SGT and SIMBA sessions may restrict our ability to compare the effectiveness of both methods fully.

Suggestions for improvement for SIMBA included insufficient time allocated for each case and lack of personalised interaction. Case simulation timings are based on the average time a student takes to complete all the questions. Undoubtedly, some students would take longer than others to answer. Therefore, any outstanding aspects of the case simulation were shared following the session's completion. It is difficult to incorporate personalised responses as not all moderators have the same clinical background. However, students can pose questions regarding the cases in the expert‐led discussion session. Given the study's focus on preclinical students, the full simulation model with history‐taking, examinations, investigations choice, diagnosis and management could not be used [17]. However, SIMBA for students was shown to help familiarise students with history‐taking and increase their knowledge in pathophysiology, which can help build confidence and prime them towards for clinical environment. Being a pilot study, the results are exploratory and provide a foundation for future large‐scale research to validate and expand on these findings.

### 6.1 Future Directions

Future directions include expanding the SIMBA for students model to medical students in clinical years. Such students would simulate history‐taking, pathophysiology, interpretation of examinations and investigations, and management and follow‐up. We will conduct the sessions with larger and more balanced cohorts to validate the findings from this pilot study. Recently, the SIMBA model has successfully been used for patient education [43]. This paves the way for future sessions where students can collaborate with patients to improve their decision‐making skills.

## 7 Conclusions

SIMBA has shown notable benefits over SGT in improving knowledge acquisition, engagement and interaction for preclinical medical and pharmacy students learning endocrinology. By leveraging accessible technology such as WhatsApp, SIMBA provides a scalable and resource‐efficient educational model that prepares students for the multifaceted demands of healthcare practice through virtual clinical simulations. SIMBA could be an innovative supplement to existing educational approaches. The six‐step conceptual framework described by Kern et al. [24] helped to define and address the needs, goals, and objectives to deliver end‐user feedback‐driven SBL. Addressing potential discomfort with technology‐based tools and self‐selection bias will be important in future iterations. Future research will focus on diverse student cohorts across multiple specialities to evaluate the potential benefits and feasibility of adopting the SIMBA model in educational settings.

## Author Contributions

**Aditya Swaminathan:** methodology, supervision, project administration, validation, writing – original draft, writing – review and editing, investigation. **Dengyi Zhou:** methodology, investigation, writing – original draft, writing – review and editing, validation, supervision. **Isabel Allison:** conceptualization, methodology, supervision, project administration, validation. **Tamzin Ogiliev:** formal analysis, writing – original draft. **Fatema Rezai:** methodology, project administration. **Georgia Morgan:** methodology, conceptualization, validation, supervision, project administration. **Haaziq Sheikh:** project administration. **Farah Abdelhameed:** project administration. **Harjeet Kaur:** project administration. **Alice Yip:** project administration. **Catherine Cooper:** project administration. **Meri Davitadze:** conceptualization, methodology, writing – review and editing, supervision, project administration. **Eka Melson:** conceptualization, methodology, validation, supervision, project administration. **Paul A. Foster:** conceptualization, methodology, project administration, supervision. **Vivek Dhir:** conceptualization, methodology, supervision, project administration. **SIMBA and CoMICs team:** project administration. **Punith Kempegowda:** conceptualization, methodology, supervision, project administration, validation, investigation, writing – review and editing.

## Ethics Statement

All methods were used per the Declaration of Helsinki. Written informed consent was obtained from all subjects. The Science, Technology, Engineering and Mathematics Committee of the University of Birmingham approved the study protocol.

## Conflicts of Interest

The authors declare no conflicts of interest.

## Supporting information


**Data S1** Image sent by moderator to participant to indicate initiation of case scenario.
**Data S2**: Questions used in SIMBA for student sessions.
**Data S3:** Thematic analysis and student responses to question: *What were the strengths of SIMBA for students and SGT?*
**Data S4:** Thematic analysis and student responses to question: *How could the SGT session have been improved?*
**Data S5:** Thematic analysis and student responses to question: *How could the SIMBA for students session have been improved?*

## Data Availability

The data that support the findings of this study are available from the corresponding author upon reasonable request.
