# Supplementary material for: SIMBA: Online Simulation for Teaching Medical Cases to Preclinical Students—A Pilot Study
Source: Clin Teach. 2025 Mar 17;22(3):e70070. doi: 10.1111/tct.70070 (PMC11913597; doi:10.1111/tct.70070)
Supplement: Supplementary file 1 — Data S1 Image sent by moderator to participant to indicate initiation of case scenario. Data S2: Questions used in SIMBA for student sessions. Data S3: Thematic analysis and student responses to question: What were the strengths of SIMBA for students and SGT? Data S4: Thematic analysis and student responses to question: How could the SGT session have been improved? Data S5: Thematic analysis and student responses to question: How could the SIMBA for students session have been improved? [file TCT-22-e70070-s001.docx]

**Supplementary 1:** Image sent by moderator to participant to indicate initiation of case scenario.

**
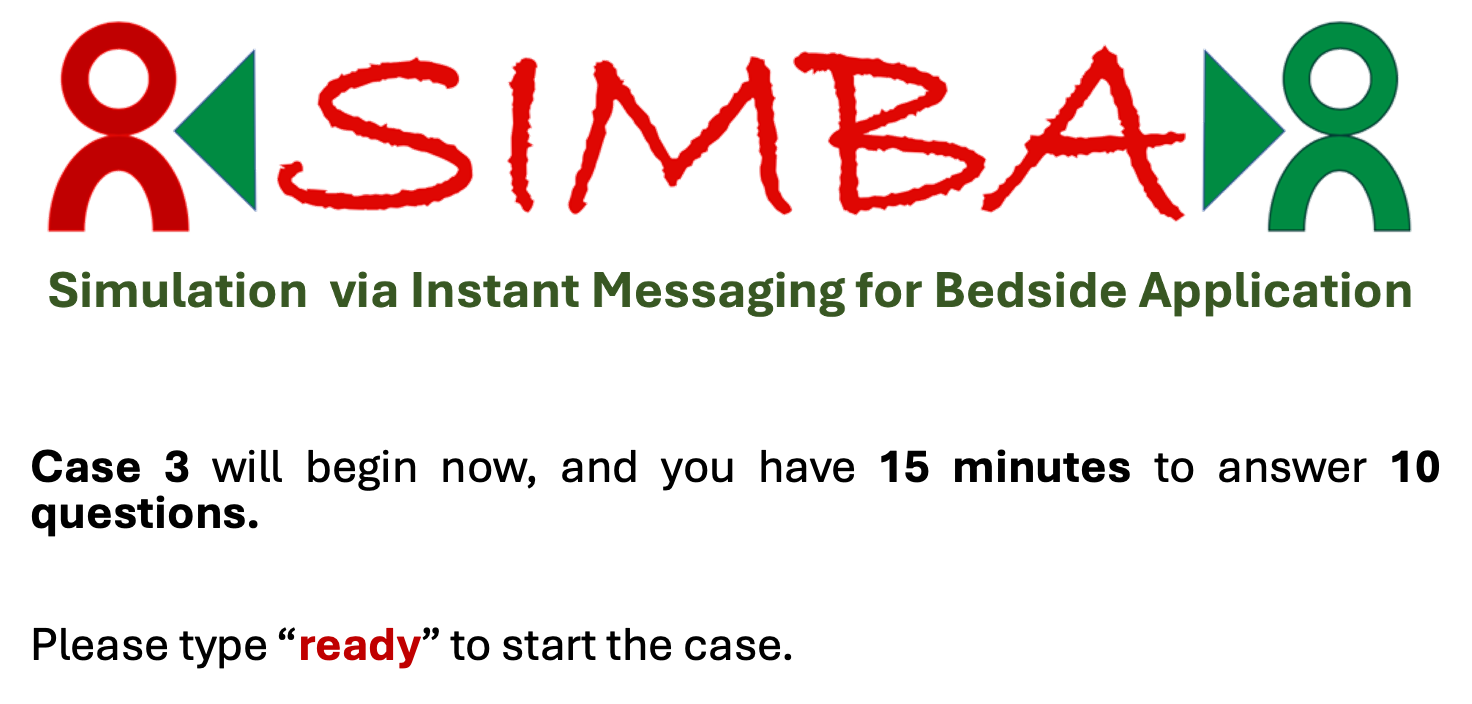
**

**Supplementary 2**: Questions used in SIMBA for Students sessions

| Year | Topic | Question | Answers | BR |
| --- | --- | --- | --- | --- |
| 20/21 | Pharm1 | A patient has been taking oral steroids for several years due to inflammatory arthritis. Which of the following signs would you be most likely to see on examination? | Exophthalmos Spider naevi **Abdominal striae** Bradycardia Malar rash | Basic Recall |
| 20/21 | Pharm1 | A patient undergoes an overnight 1mg dexamethasone suppression test. Which result would indicate Cushing’s syndrome? | **Morning cortisol <50nmol/L** Morning cortisol >50nmol/L Plasma ACTH <6pg/mL Plasma ACTH >6pg/mL  Morning cortisol <280nmol/L | Higher Order |
| 20/21 | Pharm1 | A patient presents with signs of glucocorticoid excess. Plasma ACTH is low and the CT image of adrenals comes back normal. What is a possible cause? | Pituitary adenoma Small cell lung cancer Adrenal adenoma Adrenal nodular hyperplasia **Exogenous corticosteroid use** | Higher Order |
| 20/21 | Pharm1 | Where in the adrenal gland is cortisol produced? | Zona glomerulosa Zona reticularis **Zona fasciculata** Medulla Outer capsule | Basic Recall |
| 20/21 | Pharm1 | A 38-year-old gentleman has attended for a preoperative assessment with the anaesthetist. He is due to have removal of a phaeochromocytoma. His observations show he is hypertensive at 220/110 mm Hg. What class of medication is most important to start before undergoing his surgical intervention? | **Alpha 1 blockade** Alpha 2 blockade Beta 1 blockade Beta 2 blockade Delta 1 blockade | Basic Recall |
| 20/21 | Pharm1 | Phaeochromocytoma is a tumour of which type of cells? | Kupffer cells Glandular cells Glial cells **Chromaffin cells** Beta cells | Basic Recall |
| 20/21 | Pharm1 | Where in the adrenal gland are catecholamines produced? | Zona glomerulosa Zona reticularis Zona fasciculata **Medulla** Outer capsule | Basic Recall |
| 20/21 | Pharm1 | How does noradrenaline increase blood pressure? | Stimulates aldosterone production increases reabsorption of water in the distal convoluted tubule Activates beta-1 adrenoreceptors on vascular smooth muscle **Activates alpha-1 adrenoreceptors on vascular smooth muscle** It doesn’t increase blood pressure | Basic Recall |
| 20/21 | Pharm1 | Primary adrenal insufficiency is characterised by: | Excess production of cortisol Inadequate production of aldosterone Inadequate production of cortisol Excess production of androgens **Inadequate production of cortisol and aldosterone** | Basic Recall |
| 20/21 | Pharm1 | Which of the following medications is typically prescribed in secondary adrenal insufficiency? | DHEA Fludrocortisone **Hydrocortisone** Levothyroxine Somatropin | Basic Recall |
| 20/21 | Pharm1 | What step is necessary to prevent adrenal crisis in a patient with Addison’s disease undergoing minor surgery under local anaesthetic? | IV hydrocortisone 100mg followed by continuous infusion of 200mg hydrocortisone/24h  IM hydrocortisone via emergency self-injection kit IV potassium infusion **Doubling the daily glucocorticoid dose**  No action needed | Higher Order |
| 20/21 | Pharm1 | Which of the following medications is used to replace mineralocorticoids? | Levothyroxine Hydrocortisone Dexamethasone Tetracosactrin  **Fludrocortisone** | Basic Recall |
| 20/21 | Pharm1 | Hypertension seen in primary hyperaldosteronism can be described as one of the following: | A rare example of secondary hypertension Irreversible Associated with hyperkalaemia **Resistant to the majority of anti-hypertensive medication** Due to aldosterone insufficiency | Basic Recall |
| 20/21 | Pharm1 | Where in the adrenal gland is aldosterone produced? | Zona fasciculata Zona reticularis Medulla **Zona glomerulosa** Outer capsule | Basic Recall |
| 20/21 | Pharm1 | Hypertension seen in primary hyperaldosteronism is caused by | **Excess aldosterone** Deficient aldosterone Increased stimulation of the hypothalamus-pituitary-adrenal axis Reduced stimulation of the hypothalamus-pituitary-adrenal axis Increased catecholamine release | Higher Order |
| 20/21 | Pharm1 | Which hormone directly stimulates the adrenal gland to produce aldosterone ? | Cortisol Angiotensin Renin **Angiotensin II** Angiotensinogen | Basic Recall |
| 20/21 | Pharm2 | What is the mechanism of action of SGLT 2 inhibitors? | A. Stimulates expression of insulin receptors B. Stimulates glucose-dependent insulin secretion C. Inhibits glucose reabsorption from the proximal tubule D. Inhibits gluconeogenesis E. Inhibits glucose absorption from the GI tract | Basic Recall |
| 20/21 | Pharm2 | What causes polyuria in diabetes mellitus? | A. Overactive bladder due to excess glucose acting on the nerves. B. Central obesity leading to increased pressure on the bladder C. Osmotic diuresis secondary to hyperglycaemia D. Weak pelvic floor muscles E. Frequent urine infections | Higher Order |
| 20/21 | Pharm2 | Which of the following is diagnostic of diabetes mellitus? | A. No symptoms, random plasma glucose 13 mmol/L B. No symptoms, random plasma glucose 6 mmol/L C. No symptoms, fasting plasma glucose 9 mmol/L D. Symptoms of diabetes mellitus, random plasma glucose 6 mmol/L E. Symptoms of diabetes mellitus, random plasma glucose 13mmol/L | Basic Recall |
| 20/21 | Pharm2 | Which of the following statements about metformin is true? | A. It increases gluconeogenesis B. It improves insulin sensitivity by increasing peripheral glucose uptake and utilisation C. It increases blood lipid levels D. It is a sulphonylurea E. It downregulates AMP-kinase | Basic Recall |
| 20/21 | Pharm2 | Which of the following describes the mechanism of action of sulphonylureas? | A. Stimulates insulin release from β-cells of the pancreatic islets. B. Improves insulin sensitivity by increasing peripheral glucose uptake and utilisation C. Inhibits glucose reabsorption in the proximal renal tubule D. Promotes adipogenesis and fatty acid uptake. E. Improves insulin sensitivity by increasing expression of insulin receptors. | Basic Recall |
| 20/21 | Pharm2 | Thiazolidinediones should be used cautiously in patients with comorbid: | A. Chronic Obstructive Pulmonary Disease (COPD) B. Osteoarthritis C. Hypertension D. Heart failure E. Glaucoma | Basic Recall |
| 20/21 | Pharm2 | Which of the following is an important side effect of sulphonylureas? | A. Hypoglycaemia B. Fluid retention C. Weight loss D. Gastric ulcers E. Bronchospasm | Basic Recall |
| 20/21 | Pharm2 | What is the action of GLP-1 analogues? | A. Stimulates expression of insulin receptors B. Stimulates glucose-dependent insulin secretion C. Inhibits glucose reabsorption from the proximal tubule D. Inhibits gluconeogenesis E. Inhibits glucose absorption from the GI tract | Basic Recall |
| 20/21 | Pharm2 | At what age does type 1 diabetes mellitus most commonly present? | A. 60-64 years old B. 30-34 years old C. 10-14 years old D. 80-84 years old E. 0-4 years old | Basic Recall |
| 20/21 | Pharm2 | What test might be performed to differentiate between type 1 and type 2 diabetes mellitus? | A. Blood tests for levels of islet cell antibodies, anti-insulin antibodies and/or anti-glutamic acid decarboxylase antibodies. B. Urine glucose tests C. Blood tests for HbA1c levels D. Oral glucose tolerance test (OGTT) E. Blood tests for serum insulin levels. | Basic Recall |
| 20/21 | Pharm2 | Ketosis (high blood ketone levels) in DKA is caused by: | A. Hyperglycaemia leading to break down of muscle into ketones. B. Increased reabsorption of ketones in the proximal convoluted tubule. C. Poor diet and exercise leading to excess fat, which is broken down to ketones. D. Severe insulin deficiency leading to conversion of free fatty acids to ketones in the mitochondria. E. Excess insulin leading to increased production and release of ketones. | Higher Order |
| 20/21 | Pharm2 | What is the treatment for diabetic ketoacidosis? | A. Metformin B. IV insulin C. IV furosemide D. IV glucose E. Dapagliflozin | Basic Recall |
| 20/21 | Pharm2 | Which of the following is a rapid-acting insulin analogue? | A. Actrapid B. Insuman basal C. Insuman Detemir (Levemir) D. Humulin E. Insulin Lispro (Humalog) | Basic Recall |
| 20/21 | Pharm2 | Which of the following best describes intensive insulin therapy? | A. Long-acting insulin in the evening, rapid-acting insulin with each meal. B. Rapid-acting insulin with each meal only. C. Mixed insulin twice a day. D. Mixed insulin with each meal only. E. Long-acting insulin once a day. | Basic Recall |
| 20/21 | Pharm2 | Which of the following insulin types can be administered through an insulin pump? | A. Long-acting B. Intermediate-acting C. Mixed insulin D. Rapid-acting E. Regular Human insulin | Basic Recall |
| 20/21 | Pharm3 | Which of the following correctly describes an action of PTH on the kidney? | Increases phosphate reabsorption in the proximal convoluted tubule. **Increases calcium reabsorption in the distal convoluted tubule.** Increases sodium-potassium pump activity.  Increases calcium excretion in the distal convoluted tubule. Increases reabsorption of water through aquaporin channels. | Basic Recall |
| 20/21 | Pharm3 | What is a common pattern of blood test results in primary hyperparathyroidism? | Low PTH, low calcium, raised phosphate. Low PTH, raised calcium, low phosphate Raised PTH, low calcium, raised phosphate Raised PTH, low calcium, low phosphate **Raised PTH, raised calcium, low phosphate** | Higher Order |
| 20/21 | Pharm3 | Why might levels of the active form of vitamin D (1,25-dihydroxyvitamin D) be raised in primary hyperaparathyroidism? | Raised PTH leading to increased reabsorption of 1,25-dihydroxyvitamin D from distal convoluted tubule. **Raised PTH leading to increased stimulation of 1 alpha-hydroxylase.** Raised PTH leading to increased stimulation of angiotensin converting enzyme (ACE) Raised PTH leading to inhibition of 1 alpha-hydroxylase Raised PTH leading to inhibition of angiotensin converting enzyme (ACE) | Higher Order |
| 20/21 | Pharm3 | What medication can be used to reduce PTH secretion in primary hyperparathyroidism? | Calcium carbonate Calcium gluconate **Cinacalcet** Calcitriol Calcitrex | Basic Recall |
| 20/21 | Pharm3 | Which of the following best describes the reaction that produces vitamin D in the skin? | **Photolysis of 7-dehydrocholesterol to cholecalciferol** Hydroxylation of 25-hydroxyvitamin D to 1,25-hydroxyvitamin D  Photolysis of 25-hydroxyvitamin D to cholecalciferol Hydroxylation of cholecalciferol to 25-hydroxyvitamin D Photolysis of 1,25-hydroxyvitamin D to 7-dehydrocholesterol | Basic Recall |
| 20/21 | Pharm3 | Which of the following is a possible consequence of vitamin D deficiency? | Hyperprolactinaemia  **Hyperparathyroidism** Hypernatraemia Hyperkalaemia Hypocalcaemia | Higher Order |
| 20/21 | Pharm3 | Which of the following medications is known to cause vitamin D deficiency? | Levothyroxine Naproxen Sertraline **Prednisolone** Mesalazine | Basic Recall |
| 20/21 | Pharm3 | Which form of vitamin D is replaced as part of treatment for vitamin D deficiency? | 1,25-hydroxyvitamin D 25-hydroxyvitamin D **Cholecalciferol** 7-dehydrocholesterol Vitamin D-25 hydroxylase | Basic Recall |
| 20/21 | Pharm3 | Which of the following is the first line method of restoring calcium levels in vitamin D deficiency? | Combined vitamin D and calcium supplements **Vitamin D replacement and dietary calcium intake optimisation**  Vitamin D replacement and oral bisphosphonates. Vitamin D replacement and low phosphate diet Vitamin D replacement and fluid restriction | Basic Recall |
| 20/21 | Pharm3 | Which of the following best describes the role of osteoclasts? | Formation and mineralisation of new bone matrix Hydroxylation of cholecalciferol to 1-hydroxyvitamin D Microfracture repair and mechanosensing **Bone resorption and calcium homeostasis** Bone proliferation and phosphate homeostasis | Basic Recall |
| 20/21 | Pharm3 | What is the effect of RANKL (receptor activator of nuclear factor kappa-B ligand) binding to RANK (Receptor activator of nuclear factor kappa-B)? | Initiates osteoblast differentiation and activation Inhibits osteoblast activation **Initiates osteoclast differentiation and activation** Inhibits osteoclast activation Initiates osteocyte activation | Basic Recall |
| 20/21 | Pharm3 | How do oestrogen deficiency, glucocorticoid excess and inflammation all increase the risk of osteoporosis? | Reduced absorption of vitamin D from the gut **Increased expression of RANKL on osteoblasts** Increased production of PTH Active catabolism of 1-hydroxyvitamin D Increased expression of osteoprotegerin (OPG) | Higher Order |
| 20/21 | Pharm3 | How do bisphosphonates work in treating osteoporosis? | Encourage increased bone formation Increase vitamin D formation Inhibit bone resorption Reduce calcium excretion Activate osteocyte differentiation | Higher Order |
| 20/21 | Pharm3 | What do you need to tell patients when prescribing oral bisphosphonates? | **They must take the medication on an empty stomach with large glass of water and sit/stand for 30 minutes afterwards** They must inform the DVLA and cannot drive while taking this medication This medication increases UV light sensitivity - they must cover up and wear high factor sunscreen when in the sun They must not eat foods high in calcium whilst on this medication This medication causes immunosuppression and so they should receive an annual influenza vaccination | Basic Recall |
| Year | Topic | Question | Answers | BR |
| 20/21 | Adrenal | Which of the following recurrent symptoms could be a first presentation of Cushing’s syndrome? | Recurrent syncope (fainting) Recurrent palpitations Recurrent carpal tunnel syndrome **Recurrent depressive episodes** Recurrent chest pain | Basic Recall |
| 20/21 | Adrenal | Which of the following signs is least suggestive of Cushing’s syndrome? | Moon face **Galactorrhoea** Supraclavicular fat distribution Central obesity Skin atrophy | Basic Recall |
| 20/21 | Adrenal | Which of these investigations would be most useful in differentiating between a pituitary and an ectopic cause of ACTH-dependent Cushing’s syndrome? | 24h urinary free cortisol Random plasma cortisol **48 hour high-dose dexamethasone suppression test** Overnight low-dose dexamethasone suppression test Short synacthen test | Higher Order |
| 20/21 | Adrenal | Which electrolyte abnormality can be seen in Cushing’s syndrome? | High magnesium Low phosphate **Low potassium** Low calcium Low magnesium | Basic Recall |
| 20/21 | Adrenal | A patient presents with signs of glucocorticoid excess. Plasma ACTH is low and the CT image of adrenals comes back normal. What is a possible cause? | Pituitary adenoma Small cell lung cancer Adrenal adenoma Adrenal nodular hyperplasia **Exogenous corticosteroid use** | Higher Order |
| 20/21 | Adrenal | Which of the following symptoms/signs is seen in both primary and secondary adrenal insufficiency | Hyperpigmentation Signs of hyperkalaemia Headache Vitiligo **Fatigue** | Basic Recall |
| 20/21 | Adrenal | What step is necessary to prevent adrenal crisis in a patient with Addison’s disease undergoing minor surgery under local anaesthetic? | IV hydrocortisone 100mg followed by continuous infusion of 200mg hydrocortisone/24h  IM hydrocortisone via emergency self-injection kit. Reassurance **Doubling of dose of daily glucocorticoid** No action needed | Basic Recall |
| 20/21 | Adrenal | Which of the following investigation results is NOT suggestive of primary adrenal failure? | Low cortisol High ACTH Low aldosterone Low sodium **Low renin** | Higher Order |
| 20/21 | Adrenal | Hyperpigmentation in primary adrenal failure disease is caused by | Cortisol defi **Excess ACTH** Auto-Immune reaction against the skin tissues Impairment of the skin tissues’ repair mechanism. Excess production of renin | Higher Order |
| 20/21 | Adrenal | The short synacthen test can be used to test for adrenal insufficiency. Which of the following would be a positive result? | Excessive increase in cortisol  Excessive increase in ACTH  Inadequate increase in cortisol  Inadequate increase in renin Inadquate increase in ACTH | Higher Order |
| 20/21 | Adrenal | Which of the following symptoms/signs is least suggestive of primary hyperaldosteronism? | **Hypotension**  Refractory hypertension Polyuria Polydipsia Hyporeflexia | Basic Recall |
| 20/21 | Adrenal | Hypertension seen in primary hyperaldosteronism is | A rare example of secondary hypertension Irreversible Associated with hyperkalaemia Not affected by anti-hypertensive medication Not due to excess aldosterone | Basic Recall |
| 20/21 | Adrenal | Which of the following is a sign of hypokalaemia? | Hypertonia Hyperreflexia Increased strength Cough **Muscle cramps** | Basic Recall |
| 20/21 | Adrenal | Which of the following investigation results is least suggestive of primary hyperaldosteronism? | High plasma aldosterone:renin ratio High potassium, low sodium Adrenal venous sampling showing high levels of aldosterone Abnormal adrenal glands on CT **Low plasma aldosterone:renin ratio** | Basic Recall |
| 20/21 | Adrenal | What hormone acts directly on the adrenal gland to simulate aldosterone production? | Cortisol Angiotensin Renin **Angiotensin II** Angiotensinogen | Basic Recall |
| Year | Topic | Question | Answers | BR |
| 20/21 | Adrenal | What is the most common type of adrenal tumour? | A. Phaeochromocytoma B. Cortisol-producing adenoma C. Benign endocrine inactive adenoma D. Aldosteronoma E. Adrenocortical carcinoma | Basic Recall |
| 20/21 | Adrenal | Where in the adrenal gland are androgens produced? | A. Zona fasciculata B. Zona glomerulosa C. Zona reticularis D. Medulla E. Zona granulosum | Basic Recall |
| 20/21 | Adrenal | A 38-year-old female presents to the GP complaining of absent menstrual periods (amenorrhoea) and weight gain for 6 months. She is feeling self-conscious due to increased facial hair growth (hirsutism) and facial acne. She has also noticed dark discolouration in the back of her neck, thinning hair, and hair loss from the head. Which of the following is the most likely diagnosis? | A. Hypothyroidism B. Androgen insensitivity syndrome C. Polycystic ovary syndrome D. Grave’s disease E. Type 2 diabetes mellitus | Higher Order |
| 20/21 | Adrenal | A 38 year old gentleman has attended for a preoperative assessment with the anaesthetist. He is due to have removal of a pheochromocytoma. He is usually fit and well. His observations show he is hypertensive at 220/110. What class of medication is most important to start before undergoing his surgical intervention? | A. Non-selective alpha blockade B. Alpha-2 blockade C. Beta-1 blockade D. Beta-2 blockade E. Combination of alpha-1 and beta-1 blockade | Higher Order |
| 20/21 | Adrenal | What is the most common enzyme deficiency in Congenital Adrenal Hyperplasia (CAH)? | A. 11-hydroxylase B. 17 α-hydroxylase C. 18-oxidase D. 21-hydroxylase E. C-17 lyase | Basic Recall |
| 20/21 | Adrenal | In an endocrine clinic, a male patient is diagnosed with pheochromocytoma. The consultant is interested to know more about his family history. He denies any family history of pheochromocytoma but says his father was told he had ‘a genetic condition’ and he had undergone a thyroidectomy for ‘thyroid cancer’ some years ago. What genetic condition may this patient have, supported by the family history? | A. Von Hippel Lindau syndrome B. Neurofibromatosis type 1 C. MEN2 D. MEN1 E. Succinyl Dehydrogenase complex subunit B and D | Higher Order |
| 20/21 | Adrenal | Which of the following is characteristic for polycystic ovarian morphology on ultrasound? | A. <12 follicles in both ovaries B. >10ml volume of either one or both ovaries C. Stromal hypoplasia D. Follicles 15mm in diameter E. Ovarian cysts | Basic Recall |
| 20/21 | Adrenal | Which of the following factors would suggest adrenocortical carcinoma? | A. The tumour size is 8cm B. The tumour has a low density (<10HU) on CT C. The tumour has smooth margins on imaging D. The tumour looks homogenous on imaging E. The tumour is weakly enhanced with contrast on MRI | Basic Recall |
| 20/21 | Adrenal | Which of the following is the most commonly used criteria to diagnose PCOS? | A. National Institute of Health criteria (NIH) B. The Androgen Excess and PCOS Society Criteria (AE-PCOS) C. Rotterdam criteria D. European Society of Human Reproduction and Embryology criteria (ESHRE) E. European Society of Endocrinology criteria (ESE) | Basic Recall |
| 20/21 | Adrenal | Where in the adrenal gland are catecholamines produced? | A. Zona fasciculata B. Zona glomerulosa C. Zona reticularis D. Medulla E. Zona granulosum | Basic Recall |
| 20/21 | Adrenal | Which electrolyte abnormalities may be most commonly seen in salt-wasting CAH? | A. Hyponatraemia, hypokalaemia B. Hyponatraemia, hyperkalaemia C. Hyponatraemia, normal potassium D. Hypernatraemia, hypokalaemia E. Hypernatraemia, hyperkalaemia | Higher Order |
| 20/21 | Adrenal | Which of the following is not a sign of steroid excess? | A. Low blood pressure B. Weight gain C. Abdominal striae D. Muscle weekness E. Hirsutism | Basic Recall |
| 20/21 | Adrenal | Which blood test result will be high in a patient with simple-virilising CAH? | A. Random cortisol B. Renin C. Aldosterone D. 17-hydroxyprogesterone E. Sodium | Basic Recall |
| 20/21 | Adrenal | In which way is CAH most commonly inherited? | A. Autosomal dominant B. Autosomal recessive C. Mitochondrial D. X-linked dominant E. X-linked recessive | Basic Recall |
| 20/21 | Adrenal | Which of the following drugs are most commonly used in the treatment of hirsutism in PCOS? | A. Combined Oral Contraceptive Pill B. Metformin C. 5-alpha-reductase inhibitors D. Letrozole E. Cyproterone acetate | Basic Recall |
| 20/21 | REPRO | What is the karyotype of Turner Syndrome? | 21XX 42XO **45XO** 46XY 18XX | Basic Recall |
| 20/21 | REPRO | Which of the following is not a feature of Turner Syndrome? | Congenital heart disease Cubitus valgus **Hypertrophic** **cardiomyopathy** Short stature Webbed neck | Basic Recall |
| 20/21 | REPRO | How many live births have the karyotype for Turner syndrome? | 1:500 1:1000 **1:2500** 1:5000 1:10000 | Basic Recall |
| 20/21 | REPRO | Which of the following is associated with Turner syndrome? | Addison’s disease Crohn’s disease **Hashimoto’s thyroiditis** Tetralogy of fallot Type 1 Diabetes Mellitus | Basic Recall |
| 20/21 | REPRO | A 25-year old female is having a 2-year history of irregular and unpredictable periods. She is overweight with a BMI of 32 kg/m2 and has increased facial hair growth. It is noted that her father is also overweight and developed type 2 diabetes mellitus at 45 years of age.   Which of the following is the most likely diagnosis? | Hypothyroidism Androgen Insensitivity syndrome **Polycystic ovary syndrome** Grave’s disease Type 2 diabetes mellitus | Higher Order |
| 20/21 | REPRO | Which of the following is characteristic for polycystic ovarian morphology on ultrasound? | <12 follicles in both ovaries **>10ml volume of either one or both ovaries** Stromal hypoplasia Follicles 15 mm in diameter Ovarian cysts | Basic Recall |
| 20/21 | REPRO | Which of the following is the most commonly used criteria to diagnose PCOS? | National Institute of Health criteria (NIH) The Androgen Excess and PCOS Society Criteria (AE-PCOS) **Rotterdam criteria** European Society of Human Reproduction and Embryology criteria (ESHRE) European Society of Endocrinology criteria (ESE) | Basic Recall |
| 20/21 | REPRO | Which of the following drugs are most commonly used in the treatment of hirsutism in PCOS? | **Combined Oral Contraceptive Pill** Metformin 5-alpha-reductase inhibitors Letrozole Cyproterone acetat | Basic Recall |
| 20/21 | REPRO | What chromosomal pattern would you most likely see in Klinefelter’s syndrome? | a. 45, X b. 47, XXY c. 46, XY d. 47, XYY e. 45, Y | Basic Recall |
| 20/21 | REPRO | Which blood test would be most in keeping on Klinefelter’s syndrome? | a. High testosterone, High FSH b. Low Testosterone, High FSH c. High Testosterone, High LH d. Low testosterone, Low FSH e. High Testosterone, normal LH | Higher Order |
| 20/21 | REPRO | 3. How Common is Klinefelter’s syndrome? | a. 1/1000 b. 1/2500 c. 1/100 d. 1/10,000 e. 1/500 | Basic Recall |
| 20/21 | REPRO | 4. What investigation is needed to confirm Klinefelter’s Synrome? | a. PCR test b. Serum testosterone level c. Chromosomal analysis d. Serum LH:FSH ratio e. Serum LH:testosterone level | Basic Recall |
| 20/21 | REPRO | 5. Which hormone directly causes release of Testosterone from Leydig cells? | a. FSH b. Oestrogen c. GnRH d. Dopamine e. LH | Basic Recall |
| 20/21 | REPRO | Which of the following supports a diagnosis of premature ovarian insufficiency? | High oestradiol levels  Low GnRH levels Low LH levels High testosterone levels **High FSH levels** | Basic Recall |
| 20/21 | REPRO | Which of the following is NOT a cause of premature ovarian insufficiency? | Addison’s disease Hashimoto’s disease Turner syndrome  **Polycystic ovary syndrome**  Fragile-X premutation | Basic Recall |
| 20/21 | REPRO | Which of the following is NOT a sequelae of premature ovarian insufficiency? | Joint pain  Low libido  **Visual disturbances** Vasomotor symptoms Fatigue | Basic Recall |
| 20/21 | REPRO | Why is hormone replacement treatment given in premature ovarian insufficiency? | Vasomotor symptoms Protection against cardiovascular disease Protection against osteoporosis Protection against dementia  **All of the above** | Basic Recall |
| 20/21 | REPRO | Which hormone is deficient in premature ovarian insufficiency? | **Oestradiol** Testosterone Luteinising hormone Follicle stimulating hormone Gonadotropin-release hormone | Basic Recall |
| 21/22 | Pharm1 | Which of the following symptoms is associated with Cushing’s syndrome? | Palpitations Weight loss **Moon face** Joint pain Jaundice | Basic Recall |
| 21/22 | Pharm1 | Which of the following conditions is commonly associated with Cushing’s syndrome? | Cholecystitis **Osteoporosis** Heart failure Chronic kidney disease Diabetes insipidus | Basic Recall |
| 21/22 | Pharm1 | A patient presents with signs of glucocorticoid excess. Plasma ACTH is low and the CT image of adrenals comes back normal. What is a possible cause? | Pituitary adenoma Small cell lung cancer Adrenal adenoma Adrenal nodular hyperplasia **Exogenous glucocorticosteroid use** | Higher Order |
| 21/22 | Pharm1 | Which of the following medications is used to control the hypercortisolism associated with Cushing’s syndrome? | **Metyrapone**  Carbimazole Ramipril  Propylthiouracil  Carbamazepine | Basic Recall |
| 21/22 | Pharm1 | Which of the following is a potential cause of Cushing’s syndrome? | Abuse of anabolic steroids  Adrenal gland surgery Thyroid tumour **Adrenal tumour** Obesity | Higher Order |
| 21/22 | Pharm1 | Which of the following is a recognised clinical feature of Addison’s disease? | Double vision **Weight loss** Constipation Hallucinations Polyuria | Basic Recall |
| 21/22 | Pharm1 | Which of the following is routinely measured if Addison’s disease is suspected? | 17-hydroxyprogesterone **Cortisol** Prolactin TSH Vasopressin | Basic Recall |
| 21/22 | Pharm1 | A patient with Addison’s disease is about to undergo surgery to have a total hip replacement. Which of the following actions should be taken? | Double the glucocorticoid dose **Give IV hydrocortisone**  Omit fludrocortisone 24 hours before surgery Halve the glucocorticoid dose Omit both fludrocortisone and hydrocortisone 24 hours before surgery | Higher Order |
| 21/22 | Pharm1 | Where is aldosterone produced? | Pituitary gland Zona reticularis **Zona glomerulosa** Zona fasciculata Hypothalamus | Basic Recall |
| 21/22 | Pharm1 | Which of the following conditions is associated with Addison’s disease? | Type 2 diabetes **Coeliac disease** Hypertension Multiple sclerosis Pneumocystis jirovecii | Basic Recall |
| 21/22 | Pharm1 | Which part of the adrenal gland does a phaeochromocytoma arise from? | Zona glomerulosa Zona fasciculata Zona reticularis **Adrenal medulla** Connective tissue capsule | Basic Recall |
| 21/22 | Pharm1 | Which of the following hereditary disorders is associated with phaeochromocytoma? | Huntington disease Patau syndrome **Von Hippel Lindau** Motor neuron disease Friedrich ataxia | Basic Recall |
| 21/22 | Pharm1 | Which of the following is a symptom of phaeochromocytoma? | **Tachycardia** Joint pain Dry mouth Weight gain Gynaecomastia | Basic Recall |
| 21/22 | Pharm1 | Which of the following tests is used to investigate phaeochromocytoma? | Short synacthen test **Plasma metanephrines** Salivary metanephrines Inferior petrosal sinus sampling HbA1c | Basic Recall |
| 21/22 | Pharm1 | Phaeochromocytoma is a tumour of which type of cells? | Kupffer cells Glandular cells Glial cells **Chromaffin cells** Beta cells | Basic Recall |
| 21/22 | Pharm2 | Which of the following correctly describes an action of PTH on the kidney? | Increases phosphate reabsorption in the proximal convoluted tubule. **Increases calcium reabsorption in the distal convoluted tubule.** Increases sodium-potassium pump activity.  Increases calcium excretion in the distal convoluted tubule. Increases reabsorption of water through aquaporin channels. | Basic Recall |
| 21/22 | Pharm2 | What is a common pattern of blood test results in primary hyperparathyroidism? | Low PTH, low calcium, raised phosphate. Low PTH, raised calcium, low phosphate Raised PTH, low calcium, raised phosphate Raised PTH, low calcium, low phosphate **Raised PTH, raised calcium, low phosphate** | Higher Order |
| 21/22 | Pharm2 | What medication can be used to reduce PTH secretion in primary hyperparathyroidism? | Calcium carbonate Calcium gluconate **Cinacalcet** Calcitriol Calcitrex | Basic Recall |
| 21/22 | Pharm2 | How is vitamin D produced in the skin? | **Photolysis of 7-dehydrocholesterol to cholecalciferol** Hydroxylation of 25-hydroxyvitamin D to 1,25-hydroxyvitamin D  Photolysis of 25-hydroxyvitamin D to cholecalciferol Hydroxylation of cholecalciferol to 25-hydroxyvitamin D Photolysis of 1,25-hydroxyvitamin D to 7-dehydrocholesterol | Higher Order |
| 21/22 | Pharm2 | Which of the following is a possible consequence of vitamin D deficiency? | Hyperprolactinaemia  Hyperparathyroidism Hypernatraemia Hyperkalaemia **Hypocalcaemia** | Basic Recall |
| 21/22 | Pharm2 | Which form of vitamin D is replaced as part of treatment for vitamin D deficiency? | 1,25-hydroxyvitamin D 25-hydroxyvitamin D **Cholecalciferol** 7-dehydrocholesterol Vitamin D-25 hydroxylase | Basic Recall |
| 21/22 | Pharm2 | Which of the following conditions can be associated with vitamin D deficiency? | Hypertension Type 2 Diabetes Mellitus Hyperparathyroidism **Cystic Fibrosis** Hyperthyroidism | Basic Recall |
| 21/22 | Pharm2 | Which of the following best describes the role of osteoclasts? | Formation and mineralisation of new bone matrix Hydroxylation of cholecalciferol to 1-hydroxyvitamin D Microfracture repair and mechanosensing **Bone resorption and calcium homeostasis** Bone proliferation and phosphate homeostasis | Basic Recall |
| 21/22 | Pharm2 | What is the effect of RANKL (receptor activator of nuclear factor kappa-B ligand) binding to RANK (Receptor activator of nuclear factor kappa-B)? | Initiates osteoblast differentiation and activation Inhibits osteoblast activation **Initiates osteoclast differentiation and activation** Inhibits osteoclast activation Initiates osteocyte activation | Basic Recall |
| 21/22 | Pharm2 | How do oestrogen deficiency, glucocorticoid excess and inflammation all increase the risk of osteoporosis? | Reduced absorption of vitamin D from the gut **Increased expression of RANKL on osteoblasts** Increased production of PTH Active catabolism of 1-hydroxyvitamin D Increased expression of osteoprotegerin (OPG) | Higher Order |
| 21/22 | Pharm2 | How do bisphosphonates work in treating osteoporosis? | Encourage increased bone formation Increase vitamin D formation **Inhibit bone resorption** Reduce calcium excretion Activate osteocyte differentiation | Higher Order |
| 21/22 | Pharm2 | What do you need to tell patients when prescribing oral bisphosphonates? | **They must take the medication on an empty stomach with large glass of water and sit/stand for 30 minutes afterwards** They must inform the DVLA and cannot drive while taking this medication This medication increases UV light sensitivity - they must cover up and wear high factor sunscreen when in the sun They must not eat foods high in calcium whilst on this medication This medication causes immunosuppression and so they should receive an annual influenza vaccination | Basic Recall |
| 21/22 | Pharm3 | Which of the following symptoms is associated with type 2 diabetes mellitus? | Anuria  Hypotension Anorexia **Fatigue** Tachycardia | Basic Recall |
| 21/22 | Pharm3 | Which of the following conditions can be associated with type 2 diabetes mellitus? | Coeliac Disease **Dyslipidaemia**  Hypotension Autoimmune thyroid disease Haemochromatosis | Basic Recall |
| 21/22 | Pharm3 | A 57-year-old male is reviewed in a clinic. He has no symptoms of diabetes and has a HbA1c of 7.1% (54mmol/L), what is the most appropriate next step? | Diagnose T2DM and start metformin Diagnose T2DM and start exogenous insulin therapy Do nothing **Repeat his HbA1c reading** Diagnose T2DM and give lifestyle advice only. | Basic Recall |
| 21/22 | Pharm3 | How often is HbA1c checked when someone is first diagnosed with T2DM? | Weekly Monthly Yearly Every 2 months **Every 3-6months** | Basic Recall |
| 21/22 | Pharm3 | Which of the following medications can be associated with hyperglycemia? | Furosemide Doxazosin  Spironolactone Haloperidol **Prednisolone** | Basic Recall |
| 21/22 | Pharm3 | Which of the following symptoms does type 1 diabetes mellitus most frequently present with? | **Polyuria** Adipsia Anuria Recurrent infections Anorexia | Basic Recall |
| 21/22 | Pharm3 | Which of the following conditions is associated with type 1 diabetes mellitus? | **Coeliac Disease** Hypertension Obesity Dyslipidaemia Coronary heart disease | Basic Recall |
| 21/22 | Pharm3 | Which of the following results is most likely to indicate diabetic ketoacidosis? | A. Blood glucose 23.0 mmol/L, Urinary ketones 3+, venous pH 7.41 **B. Blood glucose 23.0 mmol/L, Urinary ketones 3+, venous pH 7.21** C. Blood glucose 23.0 mmol/L, Urinary ketones 1+, venous pH 7.21 D. Blood glucose 1.3 mmol/L, Urinary ketones 3+, venous pH 7.21 E. Blood glucose 10.2 mmol/L, Urinary ketones 2+, venous pH 7.21 | Higher Order |
| 21/22 | Pharm3 | Which of the following factors may be linked to an increased risk of type 1 diabetes mellitus? | Smoking High sugar diet **Stress** Parental age at birth >35yrs Increased exercise | Basic Recall |
| 21/22 | Pharm3 | Which of the following is recommended treatment for type 1 diabetes mellitus? | Gliclazide **Subcutaneous insulin injections** Metformin Lifestyle advice Combination of oral antidiabetic drugs and subcutaneous insulin | Basic Recall |
| 21/22 | Pharm3 | What is the mechanism of action of metformin? | A. Decreasing hepatic glucose production (gluconeogenesis) and increasing intestinal absorption of glucose **B. Decreasing hepatic glucose production** (gluconeogenesis) and intestinal absorption of glucose. C. Inhibition of glucose reabsorption in the proximal renal tubule. D. Promoting adipogenesis and fatty acid uptake E. Stimulating insulin release from βcells of islets. | Basic Recall |
| 21/22 | Pharm3 | What is the mechanism of action of gliclazide? | A. Decreasing hepatic glucose production (gluconeogenesis) and increasing intestinal absorption of glucose B. Decreasing hepatic glucose production (gluconeogenesis) and intestinal absorption of glucose. C. Inhibition of glucose reabsorption in the proximal renal tubule. D. Promoting adipogenesis and fatty acid uptake **E. Stimulating insulin release from βcells of islets.** | Basic Recall |
| 21/22 | Pharm3 | What is the mechanism of action of Thiazolidinediones (glitazones)? | A. Decreasing hepatic glucose production (gluconeogenesis) and increasing intestinal absorption of glucose B. Decreasing hepatic glucose production (gluconeogenesis) and intestinal absorption of glucose. C. Inhibition of glucose reabsorption in the proximal renal tubule. **D. Promoting adipogenesis and fatty acid uptake** E. Stimulating insulin release from βcells of islets. | Basic Recall |
| 21/22 | Pharm3 | A 50-year-old man with T2DM who is currently taking metformin and gliclazide has a diabetic review. His HbA1c is 7.6% (60mmol/L), what is the most appropriate next step in management? | **A. Start a DPP-4 inhibitor** B. Start patient on insulin alongside oral antidiabetic drugs. C. Retest HbA1c without altering medication D. Swap metformin for a DPP-4 inhibitor E. Swap patient’s oral antidiabetic drugs to insulin subcutaneously. | Higher Order |
| 21/22 | Pharm3 | Which of the following is a microvascular complication of type 2 diabetes mellitus? | A. Cerebrovascular accident B. Myocardial Infarction C. Peripheral vascular disease **D. Nephropathy** E. Transient ischaemic attack | Basic Recall |
| 21/22 | Adrenal | Which of the following symptoms is associated with Cushing’s syndrome? | Palpitations Weight loss **‘Moon’ face** Joint pain Jaundice | Basic Recall |
| 21/22 | Adrenal | Which of the following conditions is commonly associated with Cushing’s syndrome? | Cholecystitis Osteoporosis Heart failure Chronic kidney disease Diabetes insipidus | Basic Recall |
| 21/22 | Adrenal | A patient presents with signs of glucocorticoid excess. Plasma ACTH is low and the CT image of adrenals comes back normal. What is a possible cause? | Pituitary adenoma Small cell lung cancer Adrenal adenoma Adrenal nodular hyperplasia **Exogenous corticosteroid use** | Higher Order |
| 21/22 | Adrenal | Which of the following medications is used to control the hypercortisolism associated with Cushing’s syndrome? | **Metyrapone**  Carbimazole Ramipril  Propylthiouracil  Carbamazepine | Basic Recall |
| 21/22 | Adrenal | Which of the following is a potential cause of Cushing’s syndrome? | Abuse of anabolic steroids  Adrenal gland surgery Thyroid tumour **Adrenal tumour** Obesity | Basic Recall |
| 21/22 | Adrenal | Which of the following is a recognised clinical feature of Addison’s disease? | Double vision **Weight loss** Constipation Hallucinations Polyuria | Basic Recall |
| 21/22 | Adrenal | Which of the following is routinely measured if Addison’s disease is suspected? | 17-hydroxyprogesterone **Cortisol** Prolactin TSH Vasopressin | Basic Recall |
| 21/22 | Adrenal | A patient with Addison’s disease is about to undergo surgery to have a total hip replacement. Which of the following actions should be taken? | Double the glucocorticoid dose **Give IV hydrocortisone**  Omit fludrocortisone 24 hours before surgery Halve the glucocorticoid dose Omit both fludrocortisone and hydrocortisone 24 hours before surgery | Higher Order |
| 21/22 | Adrenal | Where is aldosterone produced? | Pituitary gland Zona reticularis **Zona glomerulosa** Zona fasciculata Hypothalamus | Basic Recall |
| 21/22 | Adrenal | Which of the following conditions is associated with Addison’s disease? | Type 2 diabetes **Coeliac disease** Hypertension Multiple sclerosis Pneumocystis jirovecii | Basic Recall |
| 21/22 | Adrenal | Which part of the adrenal gland does a phaeochromocytoma arise from? | Zona glomerulosa Zona fasiculata Zona reticularis **Adrenal medulla** Connective tissue capsule | Basic Recall |
| 21/22 | Adrenal | Which of the following hereditary disorders is associated with phaeochromocytoma? | Huntington disease Patau syndrome **Von Hippel Lindau** Motor neuron disease Friedrich ataxia | Basic Recall |
| 21/22 | Adrenal | Which of the following is a symptom of phaeochromocytoma? | **Tachycardia** Joint pain Dry mouth Weight gain Gynaecomastia | Basic Recall |
| 21/22 | Adrenal | Which of the following tests is used to investigate phaeochromocytoma? | Short synacthen test **Plasma metanephrines** Salivary metanephrines Inferior petrosal sinus sampling HbA1c | Basic Recall |
| 21/22 | Adrenal | Which of the following would be considered as a differential diagnosis for phaeochromocytoma? | **Thyrotoxicosis** Carbon monoxide poisoning Tuberculosis Bipolar disorder Transient ischaemic attack | Higher Order |
| 21/22 | Bone | Which of the following correctly describes an action of PTH on the kidney? | Increases phosphate reabsorption in the proximal convoluted tubule. **Increases calcium reabsorption in the distal convoluted tubule.** Increases sodium-potassium pump activity.  Increases calcium excretion in the distal convoluted tubule. Increases reabsorption of water through aquaporin channels. | Basic Recall |
| 21/22 | Bone | What is a common pattern of blood test results in primary hyperparathyroidism? | Low PTH, low calcium, raised phosphate. Low PTH, raised calcium, low phosphate Raised PTH, low calcium, raised phosphate Raised PTH, low calcium, low phosphate **Raised PTH, raised calcium, low phosphate** | Higher Order |
| 21/22 | Bone | What medication can be used to reduce PTH secretion in primary hyperparathyroidism? | Calcium carbonate Calcium gluconate **Cinacalcet** Calcitriol Calcitrex | Basic Recall |
| 21/22 | Bone | How is vitamin D produced in the skin? | **Photolysis of 7-dehydrocholesterol to cholecalciferol** Hydroxylation of 25-hydroxyvitamin D to 1,25-hydroxyvitamin D  Photolysis of 25-hydroxyvitamin D to cholecalciferol Hydroxylation of cholecalciferol to 25-hydroxyvitamin D Photolysis of 1,25-hydroxyvitamin D to 7-dehydrocholesterol | Basic Recall |
| 21/22 | Bone | Which of the following is a possible consequence of vitamin D deficiency? | Hyperprolactinaemia  Hyperparathyroidism Hypernatraemia Hyperkalaemia **Hypocalcaemia** | Higher Order |
| 21/22 | Bone | Which form of vitamin D is replaced as part of treatment for vitamin D deficiency? | 1,25-hydroxyvitamin D 25-hydroxyvitamin D **Cholecalciferol** 7-dehydrocholesterol Vitamin D-25 hydroxylase | Basic Recall |
| 21/22 | Bone | Which of the following best describes the role of osteoclasts? | Formation and mineralisation of new bone matrix Hydroxylation of cholecalciferol to 1-hydroxyvitamin D Microfracture repair and mechanosensing **Bone resorption and calcium homeostasis** Bone proliferation and phosphate homeostasis | Basic Recall |
| 21/22 | Bone | What is the effect of RANKL (receptor activator of nuclear factor kappa-B ligand) binding to RANK (Receptor activator of nuclear factor kappa-B)? | Initiates osteoblast differentiation and activation Inhibits osteoblast activation **Initiates osteoclast differentiation and activation** Inhibits osteoclast activation Initiates osteocyte activation | Basic Recall |
| 21/22 | Bone | How do oestrogen deficiency, glucocorticoid excess and inflammation all increase the risk of osteoporosis? | Reduced absorption of vitamin D from the gut **Increased expression of RANKL on osteoblasts** Increased production of PTH Active catabolism of 1-hydroxyvitamin D Increased expression of osteoprotegerin (OPG) | Higher Order |
| 21/22 | Bone | How do bisphosphonates work in treating osteoporosis? | Encourage increased bone formation Increase vitamin D formation **Inhibit bone resorption** Reduce calcium excretion Activate osteocyte differentiation | Basic Recall |
| 21/22 | Bone | What do you need to tell patients when prescribing oral bisphosphonates? | **They must take the medication on an empty stomach with large glass of water and sit/stand for 30 minutes afterwards** They must inform the DVLA and cannot drive while taking this medication This medication increases UV light sensitivity - they must cover up and wear high factor sunscreen when in the sun They must not eat foods high in calcium whilst on this medication This medication causes immunosuppression and so they should receive an annual influenza vaccination | Basic Recall |
| 21/22 | Diabetes | Which of the following symptoms is associated with type 2 diabetes mellitus? | Anuria  Hypotension Anorexia **Fatigue** Tachycardia | Basic Recall |
| 21/22 | Diabetes | Which of the following conditions can be associated with type 2 diabetes mellitus? | Coeliac Disease **Dyslipidaemia**  Hypotension Autoimmune thyroid disease Haemochromatosis | Basic Recall |
| 21/22 | Diabetes | A 57-year-old male is reviewed in a clinic. He has no symptoms of diabetes and has a HbA1c of 7.1% (54mmol/L), what is the most appropriate next step? | Diagnose with T2DM and start metformin Diagnose with T2DM and start exogenous insulin therapy Do nothing **Repeat his HbA1c reading** Diagnose with T2DM and give lifestyle advice only. | Higher Order |
| 21/22 | Diabetes | How often is HbA1c checked when someone is first diagnosed with T2DM? | Weekly Monthly Yearly Every 2 months **Every 3-6months** | Basic Recall |
| 21/22 | Diabetes | Which of the following medications can be associated with hyperglycemia? | urosemide Doxazosin  Spironolactone Haloperidol **Prednisolone** | Basic Recall |
| 21/22 | Diabetes | Which of the following symptoms is associated with type 1 diabetes mellitus? | **Polyuria** Adipsia Anuria Recurrent infections Anorexia | Basic Recall |
| 21/22 | Diabetes | Which of the following conditions is associated with type 1 diabetes mellitus? | **Coeliac** **Disease** Hypertension Obesity Dyslipidaemia Coronary heart disease | Basic Recall |
| 21/22 | Diabetes | Which of the following results is most likely to indicate diabetic ketoacidosis? | A. Blood glucose 23.0 mmol/L, Urinary ketones 3+, venous pH 7.41 **B. Blood glucose 23.0 mmol/L, Urinary ketones 3+, venous pH 7.21** C. Blood glucose 23.0 mmol/L, Urinary ketones 1+, venous pH 7.21 D. Blood glucose 1.3 mmol/L, Urinary ketones 3+, venous pH 7.21 E. Blood glucose 10.2 mmol/L, Urinary ketones 2+, venous pH 7.21 | Higher Order |
| 21/22 | Diabetes | Which of the following factors may be linked to an increased risk of type 1 diabetes mellitus? | Smoking High sugar diet **Stress** Parental age at birth >35yrs Increased exercise | Basic Recall |
| 21/22 | Diabetes | Which of the following is recommended treatment for type 1 diabetes mellitus? | Oral insulin **Subcutaneous insulin injections** Metformin Lifestyle advice Combination of oral antidiabetic drugs and subcutaneous insulin | Basic Recall |
| 21/22 | Diabetes | What is the mechanism of action of metformin? | A. Decreasing hepatic glucose production (gluconeogenesis) and increasing intestinal absorption of glucose **B. Decreasing hepatic glucose production (gluconeogenesis) and intestinal absorption of glucose.** C. Inhibition of glucose reabsorption in the proximal renal tubule. D. Promoting adipogenesis and fatty acid uptake E. Stimulating insulin release from βcells of islets. | Basic Recall |
| 21/22 | Diabetes | What is the mechanism of action of gliclazide? | A. Decreasing hepatic glucose production (gluconeogenesis) and increasing intestinal absorption of glucose B. Decreasing hepatic glucose production (gluconeogenesis) and intestinal absorption of glucose. C. Inhibition of glucose reabsorption in the proximal renal tubule. D. Promoting adipogenesis and fatty acid uptake **E. Stimulating insulin release from βcells of islets.** | Basic Recall |
| 21/22 | Diabetes | What is the mechanism of action of Thiazolidinediones (glitazones)? | A. Decreasing hepatic glucose production (gluconeogenesis) and increasing intestinal absorption of glucose B. Decreasing hepatic glucose production (gluconeogenesis) and intestinal absorption of glucose. C. Inhibition of glucose reabsorption in the proximal renal tubule. **D. Promoting adipogenesis and fatty acid uptake** E. Stimulating insulin release from βcells of islets. | Basic Recall |
| 21/22 | Diabetes | A 50-year-old man with T2DM who is currently taking metformin and gliclazide has a diabetic review. His HbA1c is 7.6% (60mmol/L), what is the most appropriate next step in management? | **A. Start a DPP-4 inhibitor** B. Start patient on insulin alongside oral antidiabetic drugs. C. Retest HbA1c without altering medication D. Swap metformin for a DPP-4 inhibitor E. Swap patient’s oral antidiabetic drugs to insulin subcutaneously. | Higher Order |
| 21/22 | Diabetes | Which of the following is a microvascular complication of type 2 diabetes mellitus? | A. Cerebrovascular accident B. Myocardial Infarction C. Peripheral vascular disease **D. Nephropathy** E. Transient ischaemic attack | Basic Recall |
| 21/22 | Thyroid | 1. Which of the following symptoms is associated with hyperthyroidism? | Weight gain  Constipation Dry Skin **Tremor** Sensitivity to cold | Basic Recall |
| 21/22 | Thyroid | 2. Which of the following conditions is associated with hyperthyroidism? | Type 2 Diabetes Mellitus **Atrial fibrillation** Epilepsy Pancreatitis Chronic Kidney Disease | Basic Recall |
| 21/22 | Thyroid | What is the most likely cause of the following thyroid function test results: Free T4 - 40.6 (10-22) Free T3 - 20.2 (3.5-6.5) Serum TSH <0.01 (0.3-4.5) Anti-TPO antibodies 365 (0-59) Anti-TSH receptor antibodies 9.3 (0-1) | Hashimoto’s thyroiditis **Graves’ disease** Euthyroidism  Primary Hyperparathyroidism Thyroiditis | Higher Order |
| 21/22 | Thyroid | 1. Which of the following drugs is used to treat hyperthyroidism? | Methotrexate Levothyroxine Trimethoprim **Carbimazole** Carbamazepine | Basic Recall |
| 21/22 | Thyroid | 2. What causes Graves’ disease? | **TSH-receptor antibodies** TPO antibodies Thyroid cancer Iodine deficiency Thyroglobulin antibodies | Basic Recall |
| 21/22 | Thyroid | 1. Which of the following symptoms is associated with hypothyroidism? | Sweating Diarrhoea **Weight gain** Tremor Sensitivity to heat | Basic Recall |
| 21/22 | Thyroid | 2. Which of the following conditions is associated with hypothyroidism? | Type 2 Diabetes Mellitus  **Turner Syndrome**  Osteoarthritis Hyperparathyroidism Chronic Kidney Disease | Basic Recall |
| 21/22 | Thyroid | What is the most likely cause of the following thyroid function test results: Free T4 0.8 (10-22) Free T3 2 (3.5-6.5) Serum TSH 20.1 (0.3-4.5) Anti-TPO antibodies 312 (0-59) Anti-TSH receptor antibodies 0 (0-1) | **Hashimoto’s thyroiditis** Graves’ disease Euthyroidism Primary Hyperparathyroidism Thyroiditis | Higher Order |
| 21/22 | Thyroid | 1. Which drug is recommended to treat hypothyroidism? | Carbimazole **Levothyroxine** Thyroxidase Thiamazole Propylthiouracil | Basic Recall |
| 21/22 | Thyroid | 2. What causes Hashimoto’s thyroiditis? | **TSH-receptor antibodies** Thyroglobulin antibodies  Thyroid cancer Iodine deficiency Thyroid peroxidase (TPO) antibodies | Basic Recall |
| 21/22 | Thyroid | 1. How does thyroid function change during pregnancy? | Decrease in thyroid gland size Reduced requirement of iodine  **Increased production of thyroid hormones** Reduced thyroxine production Reduced thyroid-stimulating hormone | Basic Recall |
| 21/22 | Thyroid | 2. Why is hyperemesis gravidarum associated with increased thyroid function in pregnancy? | **Associated with raised hCG levels** Starvation stimulates TSH Antiemetics mimic TSH Reduced iodine intake  Electrolyte imbalance | Basic Recall |
| 21/22 | Thyroid | A 30-year-old pregnant female has undergone thyroid function testing.  What is the most likely cause of the following results: Free T4 24.5 (10-22) Free T3 13.2 (3.5-6.5) Serum TSH 0.2 (0.3-4.5) Anti-TPO antibodies 0 (0-59) Anti-TSH receptor antibodies 0 (0-1) | Hashimoto’s thyroiditis Graves’ disease Euthyroidism  Primary Hyperparathyroidism **Hyperthyroidism in Pregnancy** | Higher Order |
| 21/22 | Thyroid | 1. Which of the following drugs is preferred to treat Graves’ disease in the first trimester? | **Propylthiouracil** Levothyroxine Trimethoprim Carbimazole Methimazole | Basic Recall |
| 21/22 | Thyroid | 2. What is the risk to the foetus in a pregnant woman with Graves’ disease? | **Neonatal hypothyroidism** Neonatal hyperthyroidism No risk  Allergy to thyroid medication Prematurity | Higher Order |
| 21/22 | Adrenal | What is the most common type of adrenal tumour? | Phaeochromocytoma Cortisol-producing adenoma **Benign endocrine inactive adenoma** Aldosteronoma Adrenocortical carcinoma | Basic Recall |
| 21/22 | Adrenal | Which of the following factors would suggest adrenocortical carcinoma? | **The tumour size is 10 cm** The tumour has a low density (<10HU) on CT The tumour has smooth margins on imaging The tumour looks homogenous on imaging The tumour is weakly enhanced with contrast on MRI | Higher Order |
| 21/22 | Adrenal | Where in the adrenal gland are androgens produced? | Zona fasciculata Zona glomerulosa **Zona reticularis** Medulla Zona granulosum | Basic Recall |
| 21/22 | Adrenal | Which of the following is not a sign of steroid excess? | **Low blood pressure** Weight gain Abdominal striae Peripheral oedema Hirsutism | Basic Recall |
| 21/22 | Adrenal | 5. A 38 year old gentleman has attended for a preoperative assessment with the anaesthetist. He is due to have removal of a pheochromocytoma. He is usually fit and well. His observations show he is hypertensive at 220/110. What class of medication is most important to start before undergoing his surgical intervention? | **Alpha blocker** Beta blocker ACE inhibitor Calcium channel blocker Muscle relaxant | Higher Order |
| 21/22 | Adrenal | 6. In an endocrine clinic, a patient, Mr Williams, is diagnosed with pheochromocytoma. The consultant is interested to know more about Mr William’s family history. He denies any family history of pheochromocytoma but says his father was told he had ‘a genetic condition’ and he had undergone a thyroidectomy for ‘thyroid cancer’ some years ago. What genetic condition may Mr Williams have, supported by the family history? | Von Hippel Lindau syndrome Neurofibromatosis type 1 Neurofibromatosis type 2 **MEN2** Succinyl Dehydrogenase complex subunit B and D | Higher Order |
| 21/22 | Adrenal | 7. Which of the following signs might you expect to find in a patient with phaeochromocytoma? | Abdominal mass **Tremor** Bradycardia Hair loss Finger clubbing | Basic Recall |
| 21/22 | Adrenal | 8. A 42 year old female patient has symptoms suggestive of possible phaeochromocytoma. Which of the following will she have as a first line investigation as part of the diagnostic process? | PET scan **Urinary/ plasma metanephrines** Ultrasound abdo/pelvis Genetic testing Urine culture | Higher Order |
| 21/22 | Adrenal | What is the most common enzyme deficiency in Congenital Adrenal Hyperplasia (CAH)? | 11-hydroxylase 17 α-hydroxylase 18-oxidase **21-hydroxylase**  C-17 lyase | Basic Recall |
| 21/22 | Adrenal | Which blood test result will be high in a patient with simple-virilising CAH? | Random cortisol  Renin Aldosterone **17-hydroxyprogesterone** Sodium | Higher Order |
| 21/22 | Adrenal | Which electrolyte abnormalities may be most commonly seen in CAH? | Hyponatraemia, hypokalaemia **Hyponatraemia, hyperkalaemia** Hyponatraemia, normal potassium Hypernatraemia, hypokalaemia Hypernatraemia, hyperkalaemia | Higher Order |
| 21/22 | Adrenal | In which way is CAH most commonly inherited? | Autosomal dominant **Autosomal recessive** Mitochondrial X-linked dominant X-linked recessive | Basic Recall |
| 21/22 | REPRO | What is the karyotype of Turner Syndrome? | 21XX **45XO** 42XO 46XY 18XX | Basic Recall |
| 21/22 | REPRO | Which of the following is not a feature of Turner Syndrome? | Congenital heart disease Cubitus valgus **Hypertrophic cardiomyopathy** Short stature Webbed neck | Basic Recall |
| 21/22 | REPRO | Which of the following is associated with Turner syndrome? | Addison’s disease Crohn’s disease **Hashimoto’s thyroiditis** Tetralogy of fallot Type 1 Diabetes Mellitus | Basic Recall |
| 21/22 | REPRO | Which of the following investigations will be done for a patient who is being investigated for Turner’s syndrome? | CT-TAP Urinary metanephrines Abdominal X-ray **Pelvic ultrasound** Growth hormone levels | Higher Order |
| 21/22 | REPRO | A 25-year old female is having a 2-year history of irregular and unpredictable periods. She is overweight with a BMI of 32 kg/m2 and has increased facial hair growth. It is noted that her father is also overweight and developed type 2 diabetes mellitus at 45 years of age. Which of the following is the most likely diagnosis? | Hypothyroidism Androgen Insensitivity syndrome **Polycystic ovary syndrome** Grave’s disease Type 2 diabetes mellitus | Higher Order |
| 21/22 | REPRO | Which of the following drugs are most commonly used in the treatment of hirsutism in PCOS? | **Combined Oral Contraceptive Pill** Metformin 5-alpha-reductase inhibitors Letrozole Cyproterone acetate | Basic Recall |
| 21/22 | REPRO | Which of the following supports a diagnosis of premature ovarian insufficiency? | High oestradiol levels  Low GnRH levels Low LH levels High testosterone levels **High FSH levels** | Higher Order |
| 21/22 | REPRO | Which hormone is deficient in premature ovarian insufficiency? | **Oestradiol** Testosterone Luteinising hormone Follicle stimulating hormone Gonadotropin-release hormone | Basic Recall |
| 21/22 | REPRO | What chromosomal pattern would you most likely see in Klinefelter’s syndrome? | 45, X 47, XXY 46, XY **47, XYY** 45, Y | Basic Recall |
| 21/32 | REPRO | Which blood test results would be most in keeping on Klinefelter’s syndrome? | High testosterone, High FSH **Low Testosterone, High FSH** High Testosterone, High LH Low testosterone, Low FSH High Testosterone, normal LH | Higher Order |
| 21/33 | REPRO | What investigation is needed to confirm Klinefelter’s Synrome? | PCR test Serum testosterone level **Chromosomal analysis** Serum LH:FSH ratio Serum LH:testosterone level | Higher Order |
| 21/34 | REPRO | Which hormone directly causes release of Testosterone from Leydig cells? | FSH Oestrogen GnRH Dopamine **LH** | Basic Recall |

**Supplementary 3:** Thematic analysis and student responses to question: *What were the strengths of SIMBA for students and SGT?*

| Theme | Responses: SIMBA for students | Responses: SGT |
| --- | --- | --- |
| Interaction/ Engagement | Application of knowledge  *“…SIMBA is also a better reflection of knowledge application ”*  *“Application of knowledge. Opportunity to develop better understanding”* | Discussion prompt  *“Interaction with my peers and discussion helps consolidate my knowledge”*  *“clinical correlations, invitation of open discussion”* |
|  | Interactive learning  *“I liked how the questions were interactive and answers were provided instantly”*  *“More involvement and clear answers make learning clearer”* | Interactive session  *“Interactive so easier to remeber, tutors are helpful”*  *“Interactive and led by a specialist who deals with real life clinical scenarios “* |
| Session Format and Content | Case based learning  *“Good to work through it as you would in real life rather than being spoon fed it in a SGT “*  *“Case studies are useful in application of knowledge”*  *“Case experience, “* | Facilitator input  “*We are able to engage with the tutor to clarify any information but also expand with group discussion”*  *“When there is a tutor they explain concepts I don't understand well when I ask questions. …”* |
|  | Feedback and correction  *“Instant feedback"*  *“…I found it useful because after contributing our answers we were able to get some feedback answers too.”* | Group Size  *“Small groups so easy to contribute and easy to get feedback”* |

**Supplementary 4:** Thematic analysis and student responses to question: *How could the SGT session have been improved?*

| Theme | Responses |
| --- | --- |
| Session Support | Tutor Presence  *“I would prefer more facilitator led discussions for difficult concepts”*  *“I would prefer if more of the SGT's had a demonstrator like this one did as I found it really helpful”*  *“Ensure a tutor for each session as some sessions without tutor are very difficult.”* |
|  | Clear guidance on prior preparation  *“More obvious on Canvas if we need to do any prep”*  *“more guidance as to what is required before the session eg. read the sheet or answer the questions prior”* |
| Session Structure and Format | Session Feedback  *“the time it takes to get feedback could be quicker (if possible - I know professors have busy schedules) or even having a teacher involved like the diabetes session today was quite nice “*  *“Provide clear answers to the questions asked on the worksheets”* |
|  | Clear Structure and Timing  *“A clearer structure for the SGT. The explanation is always very wordy. A simple 1,2,3... step method for the SGT would be much easier to follow. …”*  *“They should be a sufficient amount of time between the lectures and SGT as most times, I do not get to finish the lecture before doing the SGT”* |

**Supplementary 5:** Thematic analysis and student responses to question: *How could the SIMBA for Students session have been improved?*

| Theme | Responses |
| --- | --- |
| Timing | During session  *“Slightly more time per case”*  *“The length of time for each case as I didn’t get to answer all of the questions.”* |
|  | In relation to curriculum  *“They could be the week after the curriculum teaching for spaced repetition. “*  *“Timing of session should coincide with end of teaching of all relevant material, so that all material has been learnt, and it can be used more as a revision/exam preparatory tool. In this case we had not covered the pharmacological aspect of diabetic treatment yet”* |
| Session Structure | Case content  *“…The questions had way too much information (although good and informative) that sometimes wasn’t that relevant to the question. “*  *“Examples of misdiagnosis situations in which the typical treatment regimen should not be carried out and what alternatives to use.”* |
|  | Moderator interaction  *“The responses are copied and pasted, the same for everyone. I think they should be personalised and address the students’ answer “*  *“Moderators could be less robotic”* |
